# Functional Multi-Locus QTL Mapping of Temporal Trends in Scots Pine Wood Traits

**DOI:** 10.1534/g3.114.014068

**Published:** 2014-10-09

**Authors:** Zitong Li, Henrik R. Hallingbäck, Sara Abrahamsson, Anders Fries, Bengt Andersson Gull, Mikko J. Sillanpää, M. Rosario García-Gil

**Affiliations:** * Department of Mathematics and Statistics, University of Helsinki, Helsinki, Finland; † Department of Forest Genetics and Plant Physiology, Swedish University of Agricultural Sciences, Umeå, Sweden; ‡ Skogforsk, Sävar, Sweden; § Department of Mathematical Sciences and Department of Biology, University of Oulu, Oulu, Finland; ** Biocenter Oulu, Oulu, Finland

**Keywords:** functional QTL mapping, wood quality traits, Scots pine, multi-locus model, shrinkage estimation

## Abstract

Quantitative trait loci (QTL) mapping of wood properties in conifer species has focused on single time point measurements or on trait means based on heterogeneous wood samples (*e.g.*, increment cores), thus ignoring systematic within-tree trends. In this study, functional QTL mapping was performed for a set of important wood properties in increment cores from a 17-yr-old Scots pine (*Pinus sylvestris* L.) full-sib family with the aim of detecting wood trait QTL for general intercepts (means) and for linear slopes by increasing cambial age. Two multi-locus functional QTL analysis approaches were proposed and their performances were compared on trait datasets comprising 2 to 9 time points, 91 to 455 individual tree measurements and genotype datasets of amplified length polymorphisms (AFLP), and single nucleotide polymorphism (SNP) markers. The first method was a multilevel LASSO analysis whereby trend parameter estimation and QTL mapping were conducted consecutively; the second method was our Bayesian linear mixed model whereby trends and underlying genetic effects were estimated simultaneously. We also compared several different hypothesis testing methods under either the LASSO or the Bayesian framework to perform QTL inference. In total, five and four significant QTL were observed for the intercepts and slopes, respectively, across wood traits such as earlywood percentage, wood density, radial fiberwidth, and spiral grain angle. Four of these QTL were represented by candidate gene SNPs, thus providing promising targets for future research in QTL mapping and molecular function. Bayesian and LASSO methods both detected similar sets of QTL given datasets that comprised large numbers of individuals.

Wood is one of the most abundant and versatile organic materials on earth. It is mainly composed of secondary cell wall consisting primarily of cellulose, lignin, and glycoprotein. Its great importance for the world economy and society has motivated the establishment of a large number of conifer breeding programs worldwide with the primary aim of increasing wood production and improving its quality (Mullin *et al.* 2011; Rosvall 2011). With respect to the genetic improvement of growth and form properties, conifer breeding has achieved notable successes. However, the costs and time required for evaluating wood properties in a large number of individuals have prevented wood quality to be incorporated into most operational breeding programs despite evidence that wood properties such as density, microfibril angle, fiber dimensions, and spiral grain harbor considerable genetic variation and moderate-to-high heritability (Gaspar *et al.* 2008; Bouffier *et al.* 2009; Gapare *et al.* 2009; Fries 2012; Hong *et al.* 2014). The assessment of wood properties is further complicated by substantial and systematic variation within trees such as early-latewood and juvenile-mature wood transitions (Zobel and Sprague 1998). Developing affordable molecular tools that could be used in, for example, early screening of tree seedlings is therefore considered a potential solution to assist wood quality improvement operationally. Moreover, access to new instruments for efficient wood characterization offers new opportunities to analyze large numbers of trees for more traits and with higher detail.

Quantitative trait loci (QTL) analysis is a technique applied to elucidate the genetic basis of quantitative traits by placing the position of putative genes underlying a complex trait onto a genetic map (Lander and Botstein 1989). QTL identification can be used to develop molecular strategies for early tree selection of economically important tree properties. QTL analyses based on anonymous DNA markers, such as amplified length polymorphisms (AFLP) or random amplified polymorphic DNA (RAPDs), identified major QTL, each explaining from 5% to 27% of the variance for pine wood traits such as density, ring width, microfibril angle, and lignin content in loblolly pine (*Pinus taeda* L.) (Knott *et al.* 1997; Kaya *et al.* 1999; Sewell *et al.* 2000, 2002; Brown *et al.* 2003), maritime pine [*Pinus pinaster* (Ait.)] (Plomion *et al.* 1996; Brendel *et al.* 2002), radiata pine (*Pinus radiata* D. Don.) (Emebiri *et al.* 1997; Kumar *et al.* 2000), and Scots pine (*Pinus sylvestris* L.) Lerceteau and Szmidt 2000; Lerceteau *et al.* 2001). More recently, high-throughput technologies have contributed to the development of a third generation of genic markers such as expressed sequence tag polymorphisms (ESTs) and single nucleotide polymorphisms (SNPs) that may increase the resolution and informativity of QTL mapping considerably. Nonetheless, only a few QTL studies have been published that have utilized ESTs and SNP in pines (Markussen *et al.* 2003; Brown *et al.* 2003; Pot *et al.* 2006). Although these studies incorporated a very limited number of gene-based markers, they have nonetheless become a useful source of candidate genes for wood related traits.

Previous QTL studies of wood properties have been limited to measurements at single time points or to trait averages of a heterogeneous wood sample (*e.g.*, stem sections, increment cores). Thus, the dynamic and systematic within-tree variation due to age and seasons was not taken into account, limiting the extent to which results could be generalized. For instance, given wood trait data from increment cores, a cumbersome process of repeated analyses on separate annual rings has been required to assess the stability of a QTL over time (Sewell *et al.* 2000, Brown *et al.* 2003, Ukrainetz *et al.* 2008). However, multiple observations of wood properties obtained by dendrochronological studies could instead be fitted to mathematical functions or curves dependent on the year of wood formation, on cambial age, or on distance from pith, a procedure called *functional QTL mapping* (Ma *et al.* 2002). The QTL analysis could then be performed on these functions, thus accounting for the systematic within-tree variability and increasing the relevance of detected QTL in comparison with analyses performed on samples specific for a certain tree age or annual ring. Functional QTL analyses have been conducted in Scots pine (Sillanpää *et al.* 2012) and *Populus* (Wu *et al.* 2003; Ma *et al.* 2002, 2004; Lin and Wu 2006) but were focused on the dynamic nature of growth rather than that of wood properties.

The functional/longitudinal QTL analysis needs to take both between- and within-individual variation into consideration. This can be implemented by a two-step multilevel approach (Gee *et al.* 2003; Heuven and Janss 2010). The phenotypic temporal trend of each individual is in the first step estimated as a curve. The curve parameters are then considered as latent traits for which marker effects are evaluated in a second independent analysis by some common QTL mapping tools. A possible alternative to such a two-step model is the linear mixed effects model (LMM) (Furlotte *et al.* 2012; Sikorska *et al.* 2012), which fits the temporal trend and estimates marker effects simultaneously in one single, albeit complex, procedure. Both two-step multilevel and LMM models are well-suited to the simultaneous effect estimation of several loci (Zeng 1994; Kao *et al.* 1999). Single locus methods, such as classical interval mapping (Lander and Botstein 1989), are simple and computationally efficient, but in cases where the sample size is small, investigated markers are numerous, and significance testing is conservative, single locus effects may suffer from severe overestimation (denoted the “Beavis effect”) (Beavis 1994; Slate 2013). Multiple locus approaches such as stepwise regression (Segura *et al.* 2012), LASSO (Tibshirani 1996), and Bayesian methods (Xu 2003; Guan and Stephens 2011) can improve in this matter. For these methods, variable selection and shrinkage estimation play key roles in avoiding oversaturated models and locus effect overestimation. In this study, we considered the LASSO variable selection for the marker effect estimation step of the multilevel approach and a Bayesian spike and slab prior approach for the linear mixed model (henceforth abbreviated as BLMM). To our best knowledge, these advanced multi-locus methods, and especially the extended functional mapping methods, have not been used in any previous QTL study for mapping wood properties. In QTL analyses, a typical procedure is to estimate the effects of the loci first, and then to quantify multi-locus association detection uncertainty or significance by applying some sort of decision rules (*e.g.*, hypothesis testing). Tests designed for LASSO and Bayesian variable selection often require some adjustments with respect to multiplicity (Scott and Berger 2010; Bühlmann *et al.* 2014). For the multilevel LASSO (mLASSO) model, one may consider a multiple-split test method (Meinshausen *et al.* 2009), a covariance test method (Lockhart *et al.* 2014), or stability selection method (Meinshausen and Bühlmann 2010). For the BLMM, which uses indicator variables, a false discovery rate control approach (Ventrucci *et al.* 2011) can be pursued.

The primary aim of this study was to determine the extent to which QTL with major effects could be detected for a set of important conifer wood properties considered as dynamic functional traits dependent on time (*i.e.*, cambial age). A secondary aim was to examine and compare two different multi-locus functional QTL mapping methods, mLASSO and BLMM on a real wood property data set, and to characterize and compare several decision rules that can be applied within the framework of mLASSO and BLMM. To achieve these two aims, functional QTL mapping was performed on a Scots pine (*Pinus sylvestris* L.) full-sib family established in a single large plot in northern Sweden using a set of AFLP and SNP molecular markers paired with phenotypic assessment of the wood of multiple annual rings in increment cores or by repeated assessments on standing trees.

## Materials and Methods

### Field test

The studied field test, named Flurkmark (S23F881485), is located 25 km north of Umeå in northern Sweden (latitude 64°02′N, longitude 20°13′E, altitude 115 m above sea level). The site is a mesic dwarf-shrub type with site index T21 (Hägglund and Lundmark 1982) and the plant material consists of 1000 offspring (F_1_) of one full-sib family. The parental (P) trees AC3065 and Y3088 were plus-trees originating from north Sweden (latitudes 65°08′N and 64°09′N, respectively) selected for the Swedish Scots pine breeding program based on their vitality, rapid growth, small branch diameters, and wide branch angles. One-yr-old seedlings were planted in 1988 using an approximate spacing of 2 × 2 m. QTL studies have previously been conducted on parts of this material with respect to other traits (Lerceteau and Szmidt 2000; Lerceteau *et al.* 2001; Sillanpää *et al.* 2012).

### Wood sampling and measurements

A subset of 286 trees was sampled for wood cores. To limit the impact of environmental variation, trees selected for sampling were all situated together in a part of the trial that exhibited the highest survival (98% in 2007) and growth. Wood samples were obtained during autumn 2004 using an electric drill combined with a 10-mm-diameter increment borer (Haglöf Sweden, AB). Samples were taken from a height of 0.8 m above the ground. They were subsequently sent to Innventia, where their properties were analyzed; the first part of the evaluations was performed and the data were made ready for the genetic studies to follow. The samples were air-dried, sawed, and polished into vertical 2 × 7 mm. From these strips, radial data profiles of wood traits (Table 1, Supporting Information, File S1) such as wood density (WD), radial fiberwidth (FWr), tangential fiberwidth (FWt), fiberwall thickness (FTh), microfibril angle (MFA), and dynamic modulus of elasticity (MOE) were acquired with the Silviscan instrument (Evans 1994, 2006). In addition to the traits assessed on increment cores, the spiral grain angle (GA) was measured at breast height (1.3 m above the ground) under bark in an extended subset of trees (492) using a nondestructive weight-and-wedge device (Chalmers University of Technology, Sweden) (File S2). Measurements were made in accordance with Hannrup *et al.* (2003) in 2006 and 2007 in the north, south, east, and west directions using the means of each year as measurement traits.

**Table 1:** List of the wood traits, their abbreviations, measurement unit, and the number of values per tree used in analysis

Traits	Abbreviation	Unit	Values/Tree
Annual ring width	RW	mm	9
Earlywood percentage	EP	%	9
Latewood percentage	LP	%	9
Mean wood density	WD	kg m^−3^	9
Earlywood density	EWD	kg m^−3^	9
Latewood density	LWD	kg m^−3^	9
Mean radial fiber width	FWr	µm	9
Earlywood radial fiber width	EFWr	µm	9
Latewood radial fiber width	LFWr	µm	9
Mean tangential fiber width*^a^*	FWt	µm	9
Mean fiberwall thickness	FTh	µm	9
Earlywood fiberwall thickness	EFTh	µm	9
Latewood fiberwall thickness	LFTh	µm	9
Microfibril angle	MFA	°	3
Modulus of elasticity	MOE	GPa	3
Grain angle*^b^*	GA	°	2

a Early and latewood components of FWt were omitted because the variation within annual rings was negligible.

b For GA, only the annual rings formed in 2006 and 2007 were studied, but the number of trees assessed (492) was considerably greater than for the other traits (286).

### Initial processing of wood property data

The first step in the evaluation of the radial profile wood trait data was to identify the interfaces between all annual rings and their parts of earlywood and latewood using methods developed by Innventia based on analysis of radial variations in wood density. Then followed calculations of the widths of each annual ring (RW) and its parts of earlywood and latewood, as well as the proportions of earlywood (EP) and latewood (LP) of each ring. Further, wood trait averages were calculated for each annual ring, for most traits averages were also calculated for their earlywood and latewood (Table 1). Then, the ring locations were manually cross-checked and the data were corrected for some errors. Finally, the annual ring data were also sorted according to the calendar year of their formation. The cambial age variation in annual rings of the same calendar year (reflecting that the trees reached the sampling height at different ages) was small, with a variation range of maximum 4 yr. The effect of cambial age was therefore assumed to be synchronous with the effect of calendar year. The nine annual rings formed during the calendar years 1995–2003 (approximately corresponding to 5–13 rings from pith) were chosen for further study because observations from those rings were reasonably complete (>95%). For MFA and MOE, the measurement resolution was low (1 observation/mm) and only allowed the study of averages of three adjacent annual rings (1995–1997, 1998–2000 and 2001–2003, respectively). In contrast, the resolution for WD, FWr, and FTh (20 observations/mm) was high enough to enable the separation of observations into earlywood and latewood components within each annual ring (Table 1) and to calculate wood trait averages for the components. There are many definitions for designating wood as earlywood and latewood (Mork 1928; Green and Worral 1964). The transition between pronounced earlywood and latewood is, however, gradual and easily influenced by site, weather, and other factors. If the annual ring is divided into two components only, then the averages for both will be influenced by all these factors, which is negative for many types of studies. To deal with this, Innventia introduced designation criteria including a third *transitionwood* component in between earlywood and latewood (Olsson 2000). Each location *i* within an annual ring was designated as earlywood or latewood depending on whether the wood density measured at that location (*WD_i_*) exceeded thresholds related to the span between the minimum wood density (*WD_min_*) and the maximum wood density (*WD_max_*) within each individual annual ring:

$$\begin{array}{l} WD_{i}\leq WD_{\min}+0.2(WD_{\max}-WD_{\min})\to i\;\text{designated as earlywood}\\ WD_{i}\geq WD_{\min}+0.8(WD_{\max}-WD_{\min})\to i\;\text{designated as latewood}\\ \;\text{otherwise }i\;\text{designated as transition wood} \end{array}$$

In the current study, the transition wood component was not studied *per se* due to its unspecific character. The data for all traits were checked with respect to normal distribution. Earlywood and latewood ratio (EP and LP) were found to deviate considerably from normality and were therefore arcsine square root–transformed (Snedecor and Cochran 1980) prior to further analysis.

### DNA extraction, marker development, and scoring

Total DNA was extracted from vegetative buds of the studied full-sib individuals. The buds were pealed and freeze-dried before being grinded and DNA was extracted using the CTAB method (Doyle and Doyle 1990).

An array of 768 single nucleotide polymorphism (SNP) markers was developed for Illumina Golden Gate assay at the University of Oulu, Department of Biology (S. T. Kujala, T. Knürr, K. Kärkkäinen, D. B. Neale, M. J. Sillanpää, and O. Savolainen, unpublished data), and it is publicly available at the Evoltree website (http://www.evoltree.eu/). During the development process, 237 SNPs were extracted from 56 gene fragments that had been sequenced in individuals of different populations by Palmé *et al.* (2008), Pyhäjärvi *et al.* (2007, 2011), and Kujala and Savolainen (2012). These gene fragments were selected mainly for their value as candidates for timing of bud set and cold tolerance. In addition, 531 SNPs from 341 other gene fragments were obtained from the *Comparative Resequencing in Pinaceae Project* (CRSP) in the laboratory of D. B. Neale, University of California at Davis (http://dendrome.ucdavis.edu/NealeLab/crsp/). The SNP genotyping was performed using GoldenGate Illumina technology at Centre National de Genotypage (CNG) in Evry, France. The automatic allele calling for each locus was accomplished with the GenCall software (Illumina, San Diego, CA).

In addition to the SNPs, several amplified length polymorphism markers (AFLP) were developed. The AFLPs were produced according to Vos *et al.* (1995). The following 15-primer enzyme combinations were used: E-act/M-cctg; E-act/M-cccg; E-act/M-ccgc; E-act/M-ccgg; E-act/M-ccag; E-acg/M-cctg; E-acg/M-cccg; E-acg/M-ccgc; E-acg/M-ccgg; E-acg/M-ccag,; E-aca/M-cctg; E-aca/M-cccg; E-aca/M-ccgc; E-aca/M-ccgg; and E-aca/M-ccag. The amplified fragments were sent to the DNA facility at Iowa State University and run on ABI3100 Genetic Analyzer. The mapping data were analyzed with GeneMarker v1.6 (SoftGenetics, State College, Pennsylvania).

A large set of 492 progeny individuals were thus genotyped using 508 AFLP markers and a small subset (91) of these individuals were also genotyped using the previously developed 768-SNP array (File S3 and File S4). The individuals of the smaller subset were all sampled for wood traits by Silviscan. Furthermore, both parents (AC3065 and Y3088) of the field trial progeny were genotyped using both SNP and AFLP markers.

### Sorting and mapping of marker data

Marker sorting and mapping were performed with all the available genotype data simultaneously, but with the aim of constructing two sorted genotype datasets, one pure AFLP dataset for the larger subset of individuals (henceforth abbreviated as the *A-set*) and one mixed SNP+AFLP dataset intended for the smaller subset (abbreviated as the *S+A set*). Because the studied full-sib family was generated by two noninbred and highly heterozygotic parents, a two-way pseudo-testcross mapping strategy was used (Grattapaglia and Sederoff 1994; Grattapaglia *et al.* 1995). Markers for which genotyping scoring success was inadequate (<80%) and poorly genotyped individuals (<70%) were excluded from further study. Even though the inclusion of 1:2:1-segregating SNPs made the construction of a consensus map possible, it was nonetheless reasonable to assume that different sets of QTL segregated within each of the unrelated and heterozygotic parents. Therefore, the marker linkage mapping was performed on maternal and paternal sections separately (see File S5 for more detailed information about the linkage mapping).

In summary, 153 AFLP markers genotyped on 455 individuals (the A dataset) and 153 AFLP and 166 SNP markers genotyped on 91 individuals (the S+A dataset) were retained in the analysis after filtering and sorting. Two-hundred fifty-one markers were distributed on 26 maternal and 24 paternal linkage groups (LGs), whereas 68 markers could not be assigned to any linkage group (unmappable).

### Multilevel functional QTL detection by LASSO variable selection

**Figure 1:**
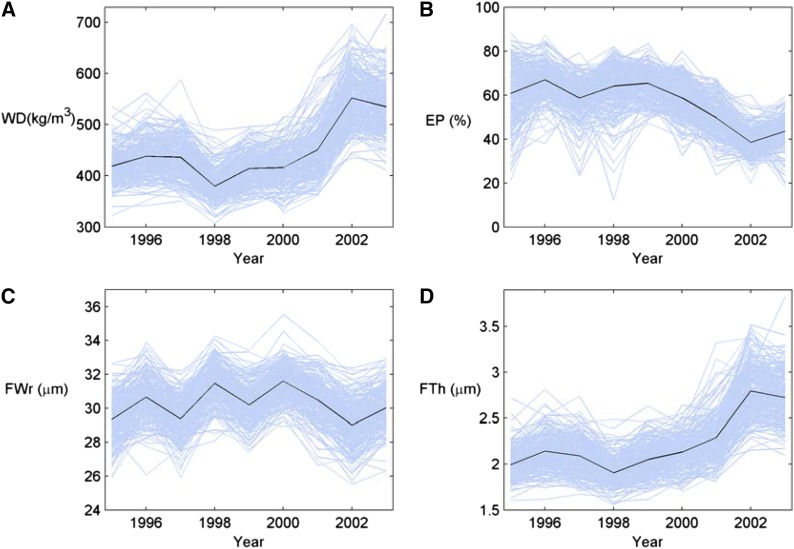
Trajectories of four wood traits by time including (A) wood density, (B) earlywood percentage, (C) radial fiberwidth, and (D) fiberwall thickness. For each trait, individual trajectories are shown in light blue lines, and the mean trajectory is shown in a black line.

Following the *multilevel* model approaches of Gee *et al.* (2003), Heuven and Janss (2010), Hurtado *et al.* (2011), and Sillanpää *et al.* (2012), we fitted our multiple phenotype measurements within each individual to a simple linear curve dependent on time *t_ik_*:

$${\text{y}}_{ik}\text{=}\mu_{i0}\text{+}\mu_{i1}t_{ik}+\varepsilon_{ik},\;\varepsilon_{ik}\overset{i.i.d.}{∼}N(0,\sigma_{i0}^{2}),$$ (1)

for individuals *i* = 1,…,*n*, and repeated measurements *k* = 1,…,*m_i_* (*m_i_* = 9 for an individual with a complete set of measurements). The least square estimates of the intercept *μ_i0_* and the slope *μ_i1_* were considered to be new *latent* traits aimed at evaluating the overall trait mean across annual rings and the trait rate of change, respectively. According to Heuven and Janss (2010), the intercepts and slopes are less correlated compared with the repeated measurements in the original scale and should have a constant variance, which would reduce the necessity to account for residual dependencies in the model. To make *μ_i0_* correspond to the biologically meaningful mean across annual rings, the time variable *t_ik_* was recoded and centralized to range from −4 to 4 instead of using the original calendar years (1995–2003). Individuals with more than five missing measurements were excluded from the analysis. Examples of the wood property development by time for a subset of traits are presented in Figure 1.

Next, to describe the association between the latent traits and the molecular markers, the following multiple linear regression models were used to map intercept (${\hat{\mu }}_{i0}$) and slope (${\hat{\mu }}_{i1}$) traits separately as:

$$\{\begin{array}{c} {\hat{\mu }}_{i0}=\alpha_{0}+\sum_{j=1}^{p}x_{ij}\beta_{j}+\alpha_{i0},\;\alpha_{i0}\overset{\text{i.i.d.}}{∼}\;N(0,\sigma_{0}^{2}),\;\\ {\hat{\mu }}_{i1}=\alpha_{1}+\sum_{j=1}^{p}x_{ij}\gamma_{j}+\alpha_{i1},\;\alpha_{i1}\overset{\text{i.i.d.}}{∼}\;N(0,\sigma_{1}^{2}),\; \end{array}$$ (2)

where *x_ij_* is the genotype value of individual *i* and marker *j* (*j* = 1,…,*p*) coded as 0 and 1 for two possible genotypes, respectively, $\alpha_{0}$ and $\alpha_{1}$ are the intercept terms, $\beta_{j}$ and $\gamma_{j}$ are the effects of marker *j*, and $\alpha_{i0}$ and $\alpha_{i1}$ are the residuals for intercept and slope traits, respectively. For the three wood traits that only have two or three phenotype assessments per tree (GA, MFA, and MOE), we performed separate LASSO analyses for each assessment. Because the ordinary least squares (OLS) method usually cannot provide accurate estimates for a number of markers near to or larger than the number of individuals assessed (the *p* > *n*-problem) (Hastie *et al.* 2009), we instead applied a popular penalized regression approach named LASSO as a marker (variable) selection method (Tibshirani 1996):

$$\{\begin{array}{c} {\left({\hat{\mu }}_{i0}-\alpha_{0}-\sum_{j=1}^{p}x_{ij}\beta_{j}\right)}^{2}+\lambda_{0}\sum_{j=1}^{p}\text{|}\beta_{j}\text{|},\\ {\left({\hat{\mu }}_{i1}-\alpha_{1}-\sum_{j=1}^{p}x_{ij}\gamma_{j}\right)}^{2}+\lambda_{1}\sum_{j=1}^{p}\text{|}\gamma_{j}\text{|}, \end{array}$$ (3)

where the penalty terms $\lambda_{0}\sum_{j=1}^{p}\left|\beta_{j}\right|$ and $\lambda_{1}\sum_{j=1}^{p}\left|\gamma_{j}\right|$, ($\lambda_{0}$, $\lambda_{1}$>0) reduce the number of markers included in the model by shrinking unimportant effects to zero. The tuning parameters $\lambda_{0}$ or $\lambda_{1}$, determine the degree of shrinkage and model sparsity. It was chosen explicitly by the 10-fold cross-validation (CV) model selection procedure (Li and Sillanpää 2012). The LASSO computation was performed by the MATLAB/R package “glmnet” (Friedman *et al.* 2010).

### Single-step functional QTL detection by Bayesian linear mixed effect model

Instead of relying on a two-step multilevel model, we may alternatively estimate the temporal trend and effects of markers simultaneously in one linear mixed effect model (BLMM). By substituting equations (2) back to equation (1), we obtain

$$\begin{array}{c} {\text{y}}_{ik}\text{=}\mu_{i0}\text{+}\mu_{i1}t_{ik}+\varepsilon_{ik}\\ =(\alpha_{0}+\sum_{j=1}^{p}x_{ij}\beta_{j}+\alpha_{i0})+(\alpha_{1}+\sum_{j=1}^{p}x_{ij}\gamma_{j}+\alpha_{i1})t_{ik}+\varepsilon_{ik}\\ =\alpha_{0}+\alpha_{1}t_{ik}+\alpha_{i0}+\alpha_{i1}t_{ik}+\sum_{j=1}^{p}x_{ij}\beta_{j}+\sum_{j=1}^{p}x_{ij}t_{ik}\gamma_{j}+\varepsilon_{ik},\;\varepsilon_{ik}\overset{i.i.d.}{∼}N(0,\sigma_{0}^{2}), \end{array}$$ (4)

which is equivalent to the longitudinal random intercept and random slope model (Fahrmeir and Kneib 2011; Furlotte *et al.* 2012; Sikorska *et al.* 2012; Wang 2012). The fixed intercept and slope parameters $\alpha_{0}$ and $\alpha_{1}$ describe the population time trends, and the random effect parameters $\alpha_{i0}$ and $\alpha_{i1}$ describe the individual specific time trends. The individual specific random intercept and slope terms are important because they: describe the deviation of individual trends over the population trend; construct a certain temporal covariance structure among phenotypes; and take the possible heterogeneity in the data caused by missing (environmental) covariates into account (Fahrmeir and Kneib 2011). We formulated a full Bayesian procedure for model inference. The fixed intercept and slope parameters were assigned with noninformative priors: $\alpha_{0}∼U(-\infty ,+\infty )$, $\alpha_{1}∼U(-\infty ,+\infty )\;$. The residual variance $\sigma_{0}^{2}$ was assigned with a Jeffreys’ noninformative prior: $p(\sigma_{0}^{2})\;\propto \frac{1}{\sigma_{0}^{2}}$. The random parameters were assigned with multivariate normal priors with common variance component $\Sigma_{2\times 2}$ over all the individuals: $\left[\alpha_{i0},\alpha_{i1}]|\Sigma_{2\times 2}∼\text{MVN}(0,\Sigma_{2\times 2}\right)$. An inverse-Wishart prior was specified for the covariance matrix: $\Sigma_{2\times 2}∼\text{Inv}-\text{Wishart}(\Psi_{2\times 2},\upsilon )$ with fixed hyperparameters $\Psi_{2\times 2}=I_{2\times 2}$ (identity matrix) and υ=1. Under such prior settings of random intercept and slope parameters, the marginal variance of *y_ik_* is $\text{Var}(y_{ik})=\sigma_{0}^{2}+\Sigma_{11}+2t_{ik}\Sigma_{12}+t_{ik}^{2}\Sigma_{22}$, and covariance between *y_ik_* and *y_ih_* (k≠h) is $\text{Cov}(y_{ik},y_{ih})=\Sigma_{11}+(t_{ik}+t_{ih})\Sigma_{12}+t_{ik}t_{ih}\Sigma_{22}$, implying that the random intercept and slope terms describe serial correlation and heterogeneity of variance for the repeated phenotype measurements in the mixed model (Weiss 2005, p. 255).

For marker effect parameters, we assigned mixture priors (or so-called spike and slab priors):

$$\beta_{j}|r_{j}∼(1-r_{j}){\text{I}}_{\left\{\beta_{j}=0\right\}}+r_{j}N(0,\sigma_{j}^{2}),\left(\text{same prior for parameter }\gamma_{j}\right)$$ (5)

Such spike and slab priors play a similar role as the *l*_1_ penalty in LASSO to perform variable selection (Habier *et al.* 2011). The binary indicator variable *r_j_* (*r_j_* = 0, 1) determines whether marker *j* should be included in the model. A Bernoulli prior was assigned to *r_j_*: $P(r_{j}|w)=w^{r_{j}}{\left(1-w\right)}^{r_{j}}$. We fixed *w* to be 0.5, so that the estimates of *r_j_* were mainly determined by the data. Furthermore, the variance parameter $\sigma_{j}^{2}$ was assigned with inverse-gamma prior: $\sigma_{j}^{2}∼\text{Inv}-\mathrm{Gamma}(a,b)$, with fixed hyperparameters *a* = 0.1, and *b* = 0.1.

For traits including MFA, MOE, and GA with two or three phenotype assessments per tree, we simplified equation (4) by including only tree-specific intercept terms but not tree-specific slope terms:

$${\text{y}}_{ik}=\alpha_{0}+\alpha_{i0}+\sum_{j=1}^{p}x_{ij}\beta_{j}+\varepsilon_{ik},\;\varepsilon_{ik}\overset{i.i.d.}{∼}N(0,\sigma_{0}^{2}),$$ (6)

In practice, a Markov Chain Monte Carlo (MCMC)-based method was used for estimating all the parameters defined in our Bayesian model. The detailed information of the MCMC sampling methods is available in the File S5.

### Quantification of uncertainty and hypothesis testing

In this study, we also treated the question of how to best quantify QTL uncertainty and significance by hypothesis testing based on LASSO and BLMM, respectively. Empirical studies (Xu 2007; Li and Sillanpää 2012) have shown that LASSO-CV tends to select many small effect markers in addition to the major QTL. The small marker effects may contribute to phenotype prediction but may not represent the foremost QTL candidates. Therefore, some post variable selection QTL inference is needed to judge which markers selected by LASSO can be declared as putative QTL with any confidence.

Constructing a test statistic for a marker under the LASSO scheme is, however, not a straightforward task, because LASSO estimates, unlike the least squares method, do not asymptotically follow any standard parameter distribution (Chatterjee and Lahiri 2011). The simplest method attempted was to first select a subset of markers by using LASSO on the whole data set, and then re-estimate the effects of those markers one-by-one with a simple regression model by the OLS method and perform the standard *t*-test (including the standard Bonferroni correction for multiple testing). This method was used as a baseline for comparisons (*p*-value is abbreviated as Single-p) and is similar to the tests of many single locus methods (Furlotte *et al.* 2012; Slate 2013).

Recently, several theoretically more solid approaches have been developed for indirectly quantifying the uncertainties of the markers selected by LASSO. We investigated the following three methods. The first is the *multiple-split test* (*p*-value is abbreviated as MST-p) of Meinshausen *et al.* (2009), which involves dividing the sample at random into two parts with equivalent number of individuals. The first half of the data are used to determine the optimal tuning parameter and to choose a subset of markers by LASSO, whereas the second half of the data are used to *t*-test those markers. For this study, the sample split procedure was repeated 100 times and the *p*-values (adjusted by Bonferroni correction) were combined to minimize the effect of random sampling of the data. The second is the *covariance test* (*p*-value is abbreviated as COV-p) proposed by Lockhart *et al.* (2014), which tests the significance of a marker entering the LASSO model and differs from the classical *t*-test of whether the marker effect equals zero. The COV-p test statistic is constructed directly based on the LASSO solution path, which is a compromise between shrinkage estimation and variable selection. Therefore, the COV-p for those selected markers should be more accurate than the *p*-values calculated by the *t*-tests. The third is the *stability selection* proposed by Meinshausen and Bühlmann (2010), involves drawing a subsample of half the number of individuals available and applying the LASSO on it to select a set of markers repeatedly (1000 times). Then, the stability selection probability (SSP) of each marker being selected was calculated and used to judge the support of QTL. Meinshausen and Bühlmann (2010) suggested a decision rule based on SSPs to control the expected number of false positives. In practice, we calculated MST-p and SSP by our own Matlab codes [please see Bühlmann *et al.* (2014) for more information about these two methods]. The COV-p was calculated by the R package “covTest” (http://cran.r-project.org/web/packages/covTest/index.html).

For the BLMM model (4), we took the advantage of the sampling of indicator variables *r_j_* defined in the prior (5). The empirical marginal posterior distribution of *r_j_*: $\hat{P}(r_{j}=1|y)$is often viewed as a posterior inclusion probability (PIP) (Guan and Stephens 2011) and has a similar interpretation as the previously mentioned SSP (Meinshausen and Bühlmann 2010). A main difference, however, is that the PIP is calculated from the MCMC samples based on the whole data without the need of repeated sub-sampling. However, the probability $\hat{P}(r_{j}=0|y)=1-\hat{P}(r_{j}=1|y)$ can be interpreted as an approximation of a local false discovery rate (LFDR) (Efron *et al.* 2001; Ventrucci *et al.* 2011) for each individual marker *j* = 1,…,*p*. We can calculate a global level posterior false discovery rate (BFDR) (Ventrucci *et al.* 2011; Heuven and Janss 2010) by combining LFDRs for a group of markers. A BFDR-based decision rule can be derived to judge significant markers to control BFDR under a certain threshold *α* (*α*=0.05) so that the multiplicity adjustments are achieved. Specifically, the LFDRs for each marker are sorted in ascending order. The average value of the LFDRs for the first *T* markers (*T* = 1,…,*p*) is defined as a BFDR for these markers. We find the highest possible value of BFDR (the average of the *T* smallest LFDRs), which is still smaller than the given threshold, and the corresponding markers are defined to be significant.

The QTL uncertainty evaluation of the results of our particular analysis was performed at two levels. If a marker attained a value less than 0.2 for *any* of the test statistics Single-p, MST-p, COV-p, or BFDR, then it was declared as a *suggestive* QTL. For the suggestive level, an SSP inclusion ratio of at least 0.66 and 0.58 was required for AFLP and SNP+AFLP datasets, respectively, which, according to the formula of Bühlmann *et al.* (2014), guaranteed the expected number of false selected markers to be less than 2. However, for a marker to be declared a *significant* QTL, requirements were stricter. For the LASSO analyses, *at least two* of the test statistics were required to show *p*-values ≤ 0.05 (Single-p, MST-p or COV-p) or an SSP inclusion ratio of at least 0.83 and 0.66 for AFLP and SNP+AFLP datasets, respectively, which guaranteed the expected number of false selected markers to be less than 1. For the Bayesian analysis, a BFDR value less than 0.05 was required. Declarations of suggestive or significant QTL were made separately for each dataset and analysis method.

We calculated the percentage of phenotypic variation explained by both suggestive and significant QTL for intercept and slope traits, respectively, as:

$$H_{\text{intercept},\text{QTL}}^{2}=\frac{\mathrm{var}(\sum_{j\in \{\text{sug}-\text{QTL}\}}x_{ij}{\hat{\beta }}_{j})}{\mathrm{var}(\mu_{i0})}\;\text{and}\;H_{\text{slope},\text{QTL}}^{2}=\frac{\mathrm{var}(\sum_{j\in \{\text{sug}-\text{QTL}\}}x_{ij}{\hat{\gamma }}_{j})}{\mathrm{var}(\mu_{i1})}$$,

respectively. Note that for the BLMM methods, we have $\mathrm{var}(\mu_{i0})\approx \mathrm{var}(\alpha_{i0})+\mathrm{var}(\sum_{j=1}^{p}x_{ij}{\hat{\beta }}_{j})$ and

$$\mathrm{var}(\mu_{i1})\approx \mathrm{var}(\alpha_{i1})+\mathrm{var}(\sum_{j=1}^{p}x_{ij}{\hat{\gamma }}_{j})$$.

## Results

From what could be observed from general trends of the studied wood material, the percentage ratio of earlywood (EP) decreased by 3.0% per year and latewood increased by 2.1% per year (Figure 1, Table 2). This trend in combination with the mean latewood density (LWD, 755 kg/m^3^) being much higher than that of earlywood density (EWD, 328 kg/m^3^) likely caused the overall ring wood density (WD) to increase by age by approximately 14.5 kg/m^3^·yr, although EWD and LWD did not show such pronounced trends *per se*. In a fashion similar to wood density, the fiber cell wall thickness (FTh) also increased (0.09 µm/yr) by increasing ring number from pith. The mean grain angle under bark (GA) at the tree age of 17–18 yr was 0.82°, implying a mild left-handed spiral grain.

**Table 2:** Time-adjusted population means, average trends by increasing tree age, and individual (phenotypic) standard deviations for means and trends of each trait

Traits	Unit	Population Mean,$E(\mu_{i0})$	Annual Ring Mean Ranges	$\text{SD}(\mu_{i0})$	Population Trend, $E(\mu_{i1})$	$\text{SD}(\mu_{i1})$
RW	mm	3.3	2.9–4.0	0.5	−0.1	0.1
EP*^a^*	%	56.3	38.6–66.6	4.4	−3.0	1.4
LP*^a^*	%	16.9	10.0–30.8	2.7	2.1	1.1
WD	kg m^−3^	448.0	380–551	27.9	14.5	6.1
EWD	kg m^−3^	327.7	307–354	18.1	−0.5	3.7
LWD	kg m^−3^	754.1	612–854	53.2	6.5	11.6
FWr	µm	30.2	29.0–31.6	0.9	∼0.0	0.2
EFWr	µm	32.7	30.8–34.2	0.8	0.3	0.2
LFWr	µm	23.1	20.1–26.4	1.1	0.5	0.3
FWt	µm	25.9	24.7–26.6	0.7	0.2	0.1
FTh	µm	2.2	1.9–2.8	0.2	0.1	<0.1
EFTh	µm	1.7	1.6–1.9	0.1	∼0.0	<0.1
LFTh	µm	3.5	2.9–4.2	0.3	0.1	0.1
MFA	°	21.2	18.9–22.3*^b^*	4.0		
MOE	GPa	10.6	9.1–13.4*^b^*	1.9		
GA	°	0.82	0.72–0.90	0.98		

a For the sake of illustration, nontransformed values are given.

b The given ranges comprise means of three adjacent annual rings rather than single annual rings.

### QTL detection for intercepts (means)

**Figure 2:**
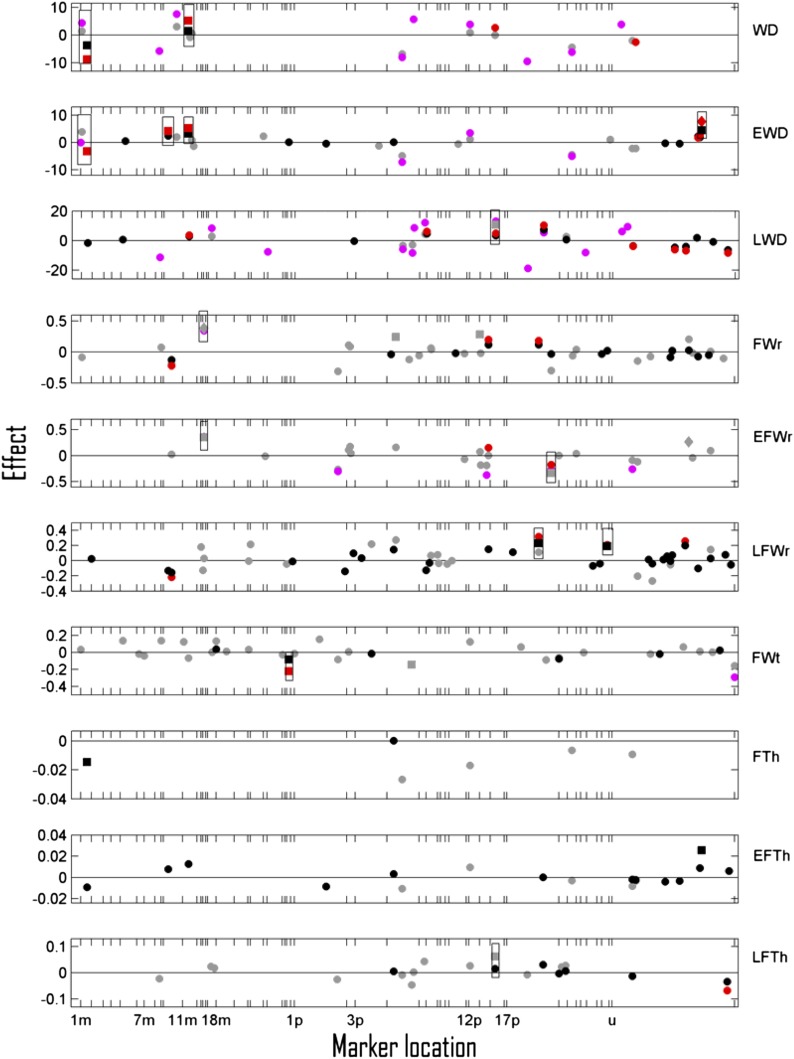
Trait intercept marker effects (*β*) for whole ring, earlywood and latewood density, whole ring radial, earlywood radial, latewood radial, whole ring tangential fiberwidths, whole ring, earlywood and latewood fiberwall thickness (WD, EWD, LWD, FWr, EFWr, LFWr, FWt, FTh, EFTh, LFTh) selected by the multilevel LASSO model in A datasets (black) and S+A datasets (gray) and of markers showing LFDR <0.5 for the Bayesian linear mixed effect model in the A datasets (red) and S+A datasets (magenta) are plotted against their estimated locations on maternal (*m*) and paternal (*p*) linkage groups (LG); 1 Morgan is approximately the length of LG 1m. Markers in section *u* were not mappable to any LG. Significant and suggestive QTL are shown as diamonds and squares, respectively, whereas all other selected markers are shown as circles. All markers in considerable linkage (recombination frequency <0.3) with a significant or suggestive QTL are highlighted within a rectangle.

**Figure 3:**
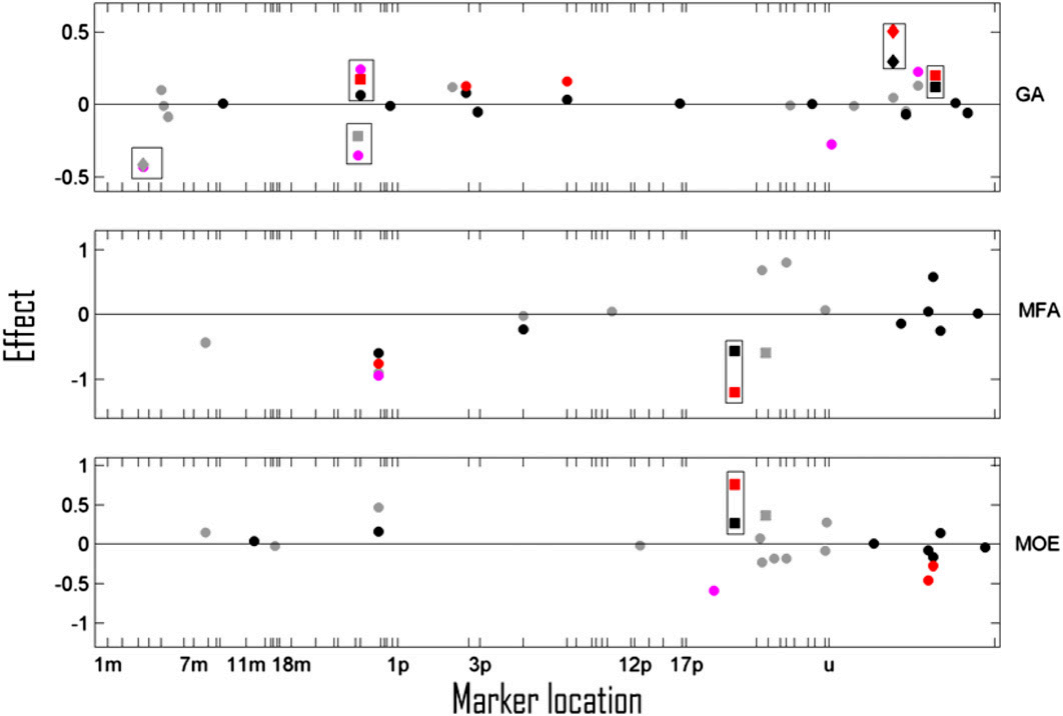
Marker effects (*β*) for wood grain angle (GA), microfibril angle (MFA), and dynamic modulus of elasticity (MOE), selected by the multilevel LASSO model in A datasets (black) and S+A datasets (gray) and of markers showing LFDR <0.5 for the Bayesian linear mixed effect model in the A datasets (red) and S+A datasets (magenta) are plotted against their estimated locations on maternal (*m*) and paternal (*p*) linkage groups (LG). 1 Morgan is approximately the length of LG 1m. Markers in section *u* were not mappable to any LG. Significant and suggestive QTL are shown as diamonds and squares, respectively, whereas all other selected markers are shown as circles. All markers in considerable linkage (recombination frequency <0.3) with a significant or suggestive QTL are framed in a rectangle. The mLASSO results are illustrated for one time point only (year 2006 for GA and the period 1998–2000 for MFA and MOE).

In total, 24 suggestive (minimum one *p*-value < 0.2) and five significant (minimum two *p*-values *<* 0.05) QTL were detected across different intercept traits (including single time point traits), datasets, and QTL mapping methods (Figure 2, Figure 3). The fractions of phenotypic intercept trait variation that was explained by QTL (*H^2^_QTL_*) ranged from zero to 0.15 (Table 3). Notably, several appreciably strong QTL were observed for the intercept of WD and EWD (three suggestive and one significant QTL) and for FWr and EFWr (three suggestive and two significant QTL) (Figure 2). These QTL explained 0.07–0.15 fractions of the phenotypic variance for the method/dataset combinations where they were observed. For EWD, both BLMM and mLASSO suggested AFLP GGG191 (unmapped) to be significant (Table 4, part A) and suggestive, respectively, in the A-set. Likewise, the SNP 0_11919_01-122 at the maternal LG 14 was indicated to have a significant association with FWr and to be suggestive for EFWr according to the mLASSO analysis performed on the S+A-set (Figure 2). The AFLP AGG142 (unmapped) was, however, only significantly associated with EFWr. The same QTL markers (suggestive or significant) were shared between EWD and WD in two instances and between FWr and EFWr in one instance. Because the QTL effect signs of whole ring and earlywood components were the same, these QTL should contribute to a positive genetic correlation between the whole ring and earlywood components for WD and FWr.

**Table 3:** The ratios of phenotypic variance

	*H^2^_QTL_* for Intercept/Means ($\mu_{i0}$)			*H^2^_QTL_* for Slopes ($\mu_{i1}$)		
Trait	mLASSO A	BLMM A	mLASSO S+A^b^	mLASSO A	BLMM A	mLASSO S+A^b^
RW	0	0	0	0	0	0
EP	0	0	0	**0.02**	0.22	0
LP	0	0	0	<0.01	0	0
WD	<0.01	0.09	0	0.01	0	0
EWD	0.02	**0.15**	0	0.01	**0.51**	0
LWD	0	0	0.01	0	0	**0.02**
FWr	0	0	**0.10**	<0.01	0	**<0.01**
EFWr	0	0	**0.07**	0	0	0.02
LFWr	0.02	0	0	0	0	0
FWt	<0.01	0.01	0.01	<0.01	0	0
FTh	<0.01	0	0	0.03	0	0
EFTh	0.01	0	0	0	0	0
LFTh	0	0	0.01	0	0	0
MFA*^a^*	0–0.01	0.02	0–0.01	**—**	**—**	**—**
MOE*^a^*	0–0.01	0.01	0–0.01	**—**	**—**	**—**
GA*^a^*	**0.03**	**0.08**	**0.06–0.07**	**—**	**—**	**—**

Ratios of phenotypic variance explained by suggestive and significant QTL jointly for the means/intercepts and slopes of all traits using mLASSO and BLMM methods for pure AFLP (A) and SNP+AFLP datasets (S+A). Trait/method/dataset combinations for which significant QTL were found are highlighted in bold.

a Ranges are given for multilevel analyses that were performed during separate time points.

b The BLMM analysis of the S+A dataset did not detect any QTL and is thus not shown.

**Table 4:** Description of significant QTL

General QTL info							Multilevel LASSO Statistics				BLMM Statistics	
QTL	Marker*^a^*	Trait	Data Set	LG*^b^*	Position (cM)	Alleles*^c^*	Multilevel Effect*^d^*	Single-p*^e^*	COV-p*^e^*	SSP*^e^*	BLMM Effect	BFDR*^e^*
Part A. QTL for trait intercepts and single time points												
**1.**	**GGG191^A^**	**EWD**	**A**	u	—	p/a	4.3 kg m^−3^	0.052′	0.235	0.688′	7.7 kg m^−3^	0.040*
1.	GGG191^A^	EWD	S+A	u	—	p/a	n.s.	—	–	—	0.5 kg m^−3^	0.651
**2.**	**0_11919_01-122^S^**	**FWr**	**S+A**	14m	11.7	C/T	0.39 µm	0.080′	0.009*	0.664*	0.35 µm	0.429
2.	—	FWr	A	14m	—	—	No AFLPs in the same LG					
**3.**	**AGG142^A^**	**EFWr**	**S+A**	u	—	p/a	0.27 µm	0.010*	<0.001*	0.682*	0.10 µm	0.624
3.	AGG142^A^	EFWr	A	u	—	p/a	n.s.	—	—	—	0.04 µm	0.690
**4.**	**TCG51^A^**	**GA***^f^*	**A**	u	—	p/a	0.30 to 0.34°	<0.001*	<0.001*	0.88–0.91*	0.51°	<0.001*
4.	TCG51^A^	GA*^f^*	S+A	u	—	p/a	0.05°	1	0.902	0.187	0.07°	0.861
**5.**	**Axs_47_502^S^**	**GA***^f^*	**S+A**	3m	40.6	A/C	−0.41 to −0.44°	0.002–0.006*	<0.001*	0.76–0.82*	−0.52°	0.227
5.	—	GA*^f^*	A	3m	—	—	No AFLPs in the same LG					
Part B. QTL for trait slopes												
**6.**	**GCG64^A^**	**EP***^g^*	**A**	u	—	p/a	0.23 y^−1^	0.006*	0.006*	0.908*	0.32 y^−1^	0.145′
6.	GCG64^A^	EP*^g^*	S+A	u	—	p/a	n.s.	—	—	—	∼0.00 y^−1^	0.978
**7.**	**TGG57^A^**	**EWD**	**A**	u	—	p/a	1.0 kg m^−3^ y^−1^	0.199′	0.215	0.712′	1.6 kg m^−3^ y^−1^	0.047*
7.	TGG57^A^	EWD	S+A	u	—	p/a	n.s.	—	—	—	0.4 kg m^−3^ y^−1^	0.691
**8.**	**2_10306_01-354^S^**	**LWD**	**S+A**	1p	474.4	A/C	3.2 kg m^−3^ y^−1^	0.071′	0.033*	0.747*	3.0 kg m^−3^ y^−1^	0.623
8.	—	LWD	A	1p	—	—	Closest AFLP (AGC141) far away (33.6 cM)					
**9.**	**0_18350_01-393^S^**	**FWr**	**S+A**	8p	0.0	A/G	−0.02 µm y^−1^	0.160′	0.035*	0.674*	−0.02 µm y^−1^	0.887
9.	—	FWr	A	8p	—	—	No AFLPs in the same LG					

Data include the name of the QTL marker, the trait and dataset where it was found, its linkage group (LG) and position within the linkage group, the alleles conferring and not conferring the effect, respectively, QTL effect estimates for multilevel LASSO and Bayesian linear mixed effect model (BLMM), and marker uncertainty quantities for Bonferroni-adjusted single ordinary least squares re-estimated *t*-test (Single-p), covariance test (COV-p), stability selection (SSP), and Bayesian global false discovery rates (BFDR), respectively. The primary QTL detections are marked in bold.

a The marker type is shown in capital superscript after the marker name: A = AFLP; S = SNP.

b m = maternal LG; *P* = paternal LG; u = unmappable.

c p/a = presence/absence.

d n.s. = not selected by LASSO.

e ′ = suggestive; * = significant.

f In case the QTL was detected for both GA assessments, effect ranges are given.

g Effects are given in the transformed scale.

For all other traits assessed with functional mapping (RW, EP, LP, LWD, LFWr, FWt, FTh, EFTh, and LFTh) QTL were fewer (in total eight suggestive) (Figure 2) and were influencing their respective traits to a relatively limited degree (*H^2^_QTL_* in the ranges 0.00–0.02) (Table 3). However, all three suggestive QTL markers for fiberwall thickness traits were also suggestive of or significant for QTL (*e.g.*, GGG191, unmapped) for the corresponding wood density traits (Figure 2). The respective QTL effects for FTh and WD had the same sign, thus suggesting a positive genetic relationship. No QTL were observed for the intercepts of annual ring width (RW), earlywood (EP), and latewood percentage (LP) (Table 3).

For the traits subjected to single-time-point LASSO analyses (GA, MFA, and MOE), the results of only one time point is shown (Figure 3) because the QTL patterns observed for the other time points were similar. Among these traits, grain angle (GA) exhibited the highest number of QTL (three suggestive and two significant), explaining up to 0.08 fraction of the phenotypic variation (Table 3). The SNP Axs_47_502 (LG 3m) and the AFLP TCG51 (unmapped) were observed to be significant QTL for GA in both the assessed years. The latter QTL exhibited the strongest overall significance values (Table 4, part A) being the only QTL significant with respect to the extremely conservative multiple-split testing method (MST-p = 0.0014 in the year 2006). This is also the reason why MST-p values were not included in Table 4. In contrast, microfibril angle (MFA) and modulus of elasticity (MOE) only exhibited two suggestive QTL each (Figure 3), which were not consistently observed across time points (not shown) and explained, at most, a 0.02 fraction of the phenotypic variation.

### QTL detection for slopes

**Figure 4:**
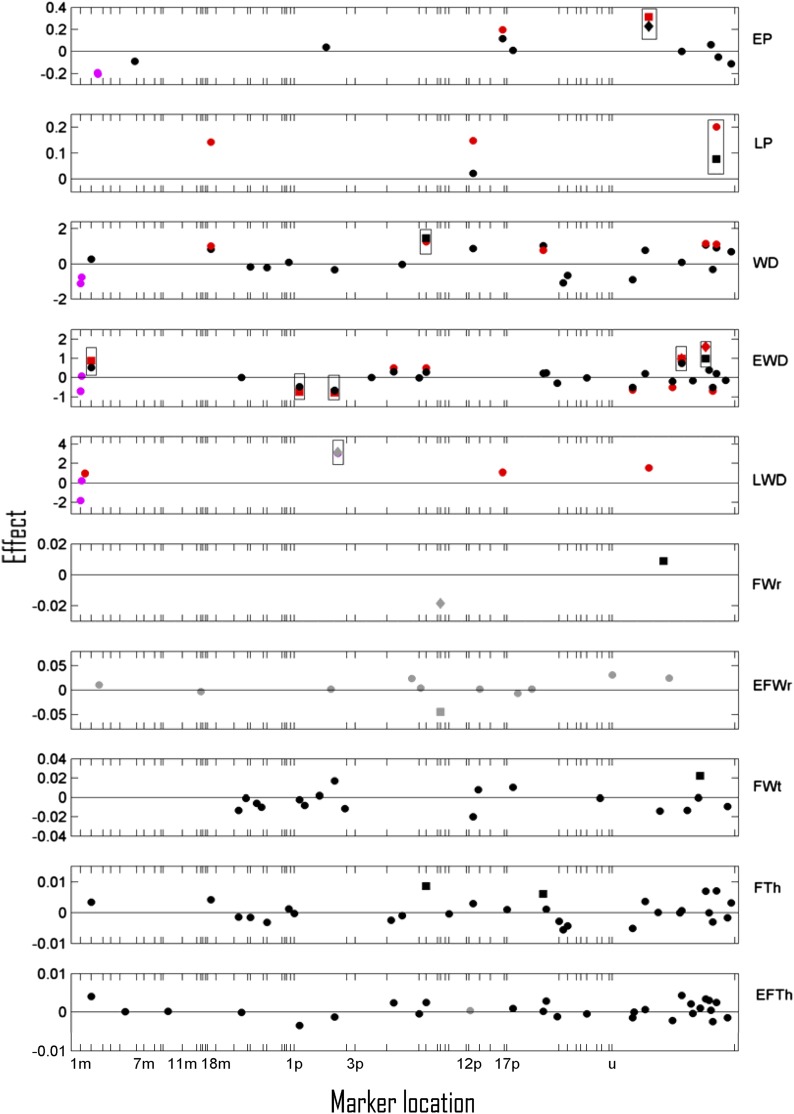
Trait slope marker effects (*γ*) for earlywood, latewood percentage ratio, whole ring, earlywood and latewood density, whole ring radial, earlywood radial, whole ring tangential fiberwidths, whole ring, and earlywood fiberwall thickness (EP, LP, WD, EWD, LWD, FWr, EFWr, FWt, FTh, EFTh) selected by the multilevel LASSO model in A datasets (black) and S+A datasets (gray) and of markers showing LFDR <0.5 for the Bayesian linear mixed effect model in the A datasets (red) and S+A datasets (magenta) are plotted against their estimated locations on maternal (*m*) and paternal (*p*) linkage groups (LG). 1 Morgan is approximately the length of LG 1m. Markers in section *u* were not mappable to any LG. Significant and suggestive QTL are shown as diamonds and squares, respectively, whereas all other selected markers are shown as circles. All markers in considerable linkage (recombination frequency <0.3) with a significant or suggestive QTL are framed in a rectangle.

For trait slopes, 11 suggestive and four significant QTL were detected (Figure 4). Interestingly, a substantial portion of the phenotypic variation for earlywood percentage (up to 0.22) (Table 3) was explained by the single QTL-AFLP GCG64 (unmapped) (Table 4, part B) significant in the mLASSO analysis and exhibiting an effect size of 13% in comparison with the trait mean (transformed scale). For the EWD slope, one significant (AFLP TGG57, unmapped) and four suggestive QTL jointly accounted for up to 0.51 fraction of the phenotypic variation.

For the slopes of LWD and FWr, one significant and one significant plus one suggestive QTL, respectively, were detected (Figure 4), but the *H^2^_QTL_* estimates for these associations were 0.02 at most (Table 3). Among those, the SNP 2_10306_01-354 (LG 1p) significantly associated with LWD nonetheless exhibited a large effect of 3.2 kg/m^3^·yr (49% in comparison to the mean), likely as a result of the considerable phenotypic variation for the LWD slope [see $\text{SD}(\mu_{i1})$in Table 2]. For all the other traits (LP, WD, EFWr, LFWr, FTh, EFTh, and LFTh), QTL findings for slopes were scarce (six suggestive QTL in total) (Figure 4) and weak (*H^2^_QTL_* ≤ 0.03) (Table 3).

### Comparing QTL mapping methods and datasets

For the statistically high-powered AFLP dataset, BLMM and mLASSO methods both appeared to detect approximately equal numbers of suggestive or significant QTL per trait (in the range of 0–5) (Figure 2, Figure 3, Figure 4). The same set of A-set markers were also frequently detected as significant QTL according to both modeling methods or were at least observed as significant for one method and suggestive for the other (Table 4). However, given that one or more QTL were detected in the A-set, the estimated proportions of explained phenotypic variances of the BLMM method (Table 3) were usually higher (0.01–0.15 and 0.22-0.51 for intercept and slope traits, respectively) than the corresponding mLASSO *H^2^_QTL_* estimates (≤0.03). Moreover, the BLMM effect estimates of the A-set were often higher than the corresponding mLASSO estimates *per se* (Figure 2, Figure 3, Figure 4). Such inconsistencies can be attributed to the different shrinkage penalties or priors involved in the two methods. LASSO assumes the shrinkage factor *λ* to be equal across all markers, whereas the Bayesian approach allows each marker to have its own individual indicator variable. Therefore, the BLMM can estimate marker effects in a more adaptive manner, *i.e.*, by shrinking less if the unaltered effect of a marker is already large.

In contrast to the observations made on the A-set, BLMM did not even detect a single suggestive QTL in the statistically low-powered SNP+AFLP dataset, whereas the mLASSO method detected similar numbers of suggestive or significant QTL in both the S+A-sets and A-sets (Table 3). To further dissect potential causes of this discrepancy, we conducted additional analyses combining the Bayesian spike and slab approach and BFDR testing with the two-step method. Interestingly, this two-step Bayesian spike and slab model was, similar to mLASSO, able to detect a number of QTL even in the S+A-set (not shown), implying that the previously observed discrepancies reflect differences in the modeling procedure (one-step *vs.* two-step) rather than statistical approach (LASSO *vs.* Bayesian). The Bayesian spike and slab model and LASSO should thus have similar power for detecting QTL given that the same model procedure is pursued.

### Comparison between decision-making/hypothesis testing methods

To have an approximate comparison between the testing methods, we simply screened all the traits and counted the total number of significant or suggestive QTL for the large A- and small S+A datasets, respectively (for LASSO analyses of single time-point traits, only the counts for GA measured in 2006 and MFA and MOE observed in 1998–2000 were included). For the A-set, the ranking of testing methods based on the greatest total number of detected significant and suggestive QTL fell in the order BFDR > Single-p > SSP > COV-p > MST-p (Table 5). For the S+A-set with fewer numbers of individuals, the ranking order was COV-p > Single-p > SSP > MST-p > BFDR. The results for the BFDR of the extra two-step Bayesian analysis were quite similar to that of the Single-p and SSP tests of the mLASSO analysis (not shown).

**Table 5:** Counts of significant or suggestive QTL summed over all the traits for AFLP data and SNP+AFLP data

Data Type	Multilevel LASSO				BLMM
	Single-p	MST-p	COV-p	SSP	BFDR
Counts of significant QTL					
AFLP	2	1	4	2	3
SNP+AFLP	2	0	5	4	0
Total number of suggestive or significant QTL					
AFLP	17	1	12	14	18
SNP+AFLP	7	0	12	5	0

## Discussion

To the knowledge of the authors, this study presents the first functional/longitudinal QTL analysis of a conifer wood properties dataset with repeated phenotype measurements made possible by use of efficient measurement methods. Such analyses, so far, have only been performed for growth trajectories (Sillanpää *et al.* 2012). Compared with earlier QTL studies, functional mapping analyses utilize all the longitudinal data for a trait simultaneously and may better account for temporal trends and correlation structures across years. It can thus detect QTL that are stable over time (*i.e.*, the QTL associated with intercept traits) with greater statistical evidence, but it can also identify QTL that interact with time (*e.g.*, the QTL associated with slope traits) (Wu and Lin 2006). Summary statistics on the wood properties of the studied Scots pine full-sib family (Figure 1, Table 2) showed levels and trends well in line with expectations of young Scots pines (compare with Fries 2012) in the cambial age range of approximately 5–13 yr. The tree age of this material (17 yr) is also considered to be appropriate for assessment and selection in breeding (Rosvall 2011), making the results of this study also relevant from a breeding point of view. Given the cambial age range of the increment cores, our wood trait slope parameters should illustrate the rate of the biological transition from juvenile to mature wood by increasing cambial age. Previous research on wood properties has indicated wood trait radial trends of mature trees to be best-fitted by exponential or logistic models (Burdon *et al.* 2004). However, the nine investigated annual rings only partly cover the juvenile-to-mature wood transition phase, which in Scots pine may extend over a time period as long as 20 yr (Hannrup *et al.* 2000). Moreover, wood properties are also known to be strongly influenced by annual environmental fluctuations such as climate (Feng *et al.* 2012), which could have caused the deviations from linearity observed in Figure 1 as readily as increasing cambial age. Therefore, a simple linear function was chosen for the functional mapping of this study despite the minor nonlinearities observed for the trait trajectories.

The functional mapping of this study moreover investigated two advanced but closely related multi-locus model approaches: a multilevel LASSO model (mLASSO) and a newly developed Bayesian linear mixed model (BLMM). The use of multi-locus models with shrinkage of QTL marker effects (LASSO and BLMM) could act as a safeguard against the systematic effect overestimation associated with single-locus models paired with conservative multiple significance testing (Beavis 1994; Slate 2013). Compared with the approach of Sillanpää *et al.* (2012), our new BLMM approach can be more efficient because of the prior conjugacy and its fast implementation in Matlab. The BLMM is also closely connected to many other Bayesian functional mapping approaches such as those of Yang and Xu (2007) and Li and Sillanpää (2013). However, those studies modeled the marker effects as nonlinear curves over time, whereas the marker effects of our study simply constitutes parameters that describe a linear trend *per se*. Another difference is that they assumed the effects of the same marker on slope and intercept to be correlated in the priors, whereas we assume them to be independent. The principle of the two-step mLASSO of this study is similar to QTL mapping approaches introduced by Gee *et al.* (2003), Heuven and Janss (2010), and Hurtado *et al.* (2011), even though they used effect estimation methods other than LASSO.

### QTL observed for the intercept of important wood traits

Results from the wood intercept trait analyses, aimed at detecting QTL stable over a specified period of time, largely indicated zero to five QTL per trait explaining a small fraction of the phenotypic variation and with limited effects on the traits. This observation agrees with many published conifer QTL and association mapping studies (Brown *et al.* 2003; Pot *et al.* 2006; González-Martínez *et al.* 2007; Ukrainetz *et al.* 2008; Dillon *et al.* 2010; Beaulieu *et al.* 2011) and, given that only major QTL usually are detected, it suggests wood intercept traits to be largely polygenic. In our study, EWD and FWr exhibited a greater number of appreciably strong QTL stable over time in comparison with the latewood proportion of those traits or to other wood traits (Figure 2, Table 3). However, the three QTL markers significant for EWD, FWr, and EFWr exhibited individual effects in a range of 0.8%–2.3% in relation to the overall population mean of the respective traits. Consequently, selection assisted by markers developed from these QTL would likely not achieve any dramatic improvements if used in isolation. The observations of numerous and stable EWD QTL agree with previous results where separate annual ring QTL analyses were performed on the wood of a full-sib family (Brown *et al.* 2003) and a three-generation pedigree (Sewell *et al.* 2000) of loblolly pine. Also, Ukrainetz *et al.* (2008) detected several QTL for both EWD and LWD in several separate annual ring QTL analyses on eight full-sib families of Douglas fir [*Pseudotsuga menziesii* (Mirb) Franco var *menziesii*].

The observed tendency of WD and FWr to exhibit sets of QTL markers similar to those of EWD and EFWr, respectively, indicates genetic regulation of the earlywood component of the annual ring to be highly influential on properties of the annual ring as a whole. This is not very surprising because earlywood constituted the major component in most annual rings studied (Table 2). Similarly, the observation of three QTL markers suggestively or significantly associated with both fiberwall thickness and wood density traits is consistent with the strong phenotypic and genetic correlations repeatedly observed between wood density and the thickness of the tracheid cell walls (for examples of Scots pine, see Fries 2012; Hong *et al.* 2014). The apparent universal applicability of these correlations therefore suggests that the QTL colocalizations we observed for these trait pairs (WD/EWD, FWr/EFWr, and WD/FTh) more likely stem from pleiotropy than from close linkage.

Also, for spiral grain angle, which is a trait closely connected to the twist propensity of sawed timber during drying (Dinwoodie 2000; Warensjö and Rune 2004), a number of influential QTL (two significant and three suggestive) were observed (Figure 3). To the knowledge of the authors, no QTL for grain angle have been published for any conifer species. In contrast to the estimated marker effects of the intercept traits, effects of the significant GA QTL (0.30°–0.52°) (Table 4, part A) indicate that an appreciable reduction of GA could be achieved by marker-assisted selection in the material of this study and potentially improve the shape stability of small sawed timber (Hallingbäck *et al.* 2010). However, it must still be cautioned that the GA measurements were taken directly beneath the bark at breast height during two consecutive growth seasons (cambial age range of 12–14 yr) and the detected QTL thus may not be stable over a wider range of cambial ages. For other traits observed at few time points such as MFA and MOE, only a limited set of suggestive QTL were observed.

### QTL observed for the slopes of important wood traits

The slope of wood traits over cambial ages or the rate of juvenile-to-mature wood transition has, to our knowledge, never been included in any published QTL or association mapping analyses, even though one association mapping study of radiata pine (Dillon *et al.* 2010) considered the transition in a different manner. They regarded the juvenile-to-mature wood transition as a discrete phase change whose timing could be predicted using latewood density as an indicator trait (Gapare *et al.* 2006), and we have rather dissected the dynamics of the transition process itself. In the results of this study, it was notable that large portions of the phenotypic variation for the slopes of earlywood percentage and earlywood density were explained by a limited number of suggestive and significant QTL (Table 3 and Figure 4) indicating the existence of major effect loci. Also, the effect of the QTL marker significantly associated with the slope of LWD (SNP 2_10306_01-354) (Table 4, part B) was notably large despite the low corresponding *H^2^_QTL_* estimates. The results for EP and LWD are particularly intriguing because these traits are well-known to exhibit decreasing and increasing trends, respectively (Figure 1B, Table 2) as a result of the wood maturation process (Zobel and Sprague 1998; Burdon *et al.* 2004). Taking the significant EP QTL (GCG64) (Table 4, part B) as an example and assuming conditions similar to those of this study, trees with the *band present* genotype (heterozygote) are predicted to decrease their EP from 56.3% at cambial age 9 to 42.2% at age 14 (5 yr later), whereas trees with the *band absent* genotype (homozygote) would decrease their EP even faster (from 56.3% to 40.3%) during the same time period. Admittedly, this calculation example may be oversimplistic and one should remember that QTL observed in a single full-sib family are usually not generalizable to other families, populations, or environments. However, the results of this study nonetheless illustrate potential to regulate the speed of certain aspects of wood maturation by marker-assisted selection.

### Comparison between QTL mapping methods and decision rules

As shown in *Materials and Methods*, the mLASSO model and BLMM model are in principle closely connected approaches. The mLASSO model is a two-step approach because it estimates the temporal trends and marker effects on those trends separately, whereas the BLMM is a one-step approach estimating the temporal trends and marker coefficients simultaneously. Furthermore, in mLASSO, the intercept and slope latent parameters are treated independently. However, in BLMM, intercepts and slopes are jointly analyzed in a bivariate model, so the dependency between them may be more properly taken care of (Piepho *et al.* 2012). In general, it is believed that BLMM should be a more precise approach for analyzing this type of longitudinal data, and that the mLASSO model can sometimes be used as an approximation to BLMM (Sikorska *et al.* 2012). In our work, mLASSO and BLMM detected similar sets of QTL under conditions of higher statistical power (the A-set with data for at least 250 individuals), whereas under conditions of low statistical power (S+A-set with data for less than 100 individuals regardless of the trait) only the mLASSO was able to detect any QTL (Figure 2, Figure 3, Figure 4 and Table 4). Supported by results of additional two-step Bayesian analyses, it is possible that the one-step BLMM, due to the simultaneous estimation of a higher number of parameters than the mLASSO, provided estimates with larger variances and lost power to detect QTL when sample sizes were insufficient. However, it also cannot be excluded that the small sample size of the S+A-set combined with the approximative behavior of the two-step methods (like mLASSO) could have caused a greater-than-expected number of falsely detected QTL. In any case, the inconsistency between BLMM and mLASSO at low statistical power suggests that QTL observed in the S+A dataset should be interpreted cautiously.

Another methodological objective of this study was to evaluate the performance of several hypothesis-testing or uncertainty assessment methods for QTL identification. Regarding the mLASSO framework, the Single-p is very similar to many single locus hypothesis testing methods commonly used, whereas MST-p, COV-p, and SSP are more specifically designed for multi-locus LASSO methods. One common problem regarding these tests is their inconsistency with penalized estimates of marker effects obtained at a specific value of tuning parameter *λ* in LASSO. The development of more reliable LASSO test statistics is still an ongoing research topic (Lockhart *et al.* 2014). Under the BLMM framework, it is possible to derive a BFDR-based decision rule by relying on a single MCMC simulation without any extra effort such as re-sampling. Given the results of this study, one can conclude that MST-p was much more conservative than all other methods. This was expected because MST-p relies on a stricter assumption than the others (Bühlmann *et al.* 2014). In future research, simulation studies are needed to perform more intensive comparisons between the BLMM and the mLASSO approaches and between their associated significance testing methods.

### Potential links between QTL and candidate genes

Among the nine significant QTL observed in this study, four were linked to SNP markers (Table 4), which would enable speculations about the potential function of the candidate genes from which the SNPs were developed. One should, however, note that none of these QTL SNPs were mapped in the proximity to any of the AFLP markers, which could have verified the QTL in the statistically high-powered A-set. Moreover, because conifer genomes are very large, the genetic linkage within our full-sib family likely extends over numerous genes that, due to the limited statistical power of the S+A-set, all could be considered as alternative loci for the QTL. Consequently, the interpretations made here need to be taken with caution.

The significant SNP-QTL for the intercept of FWr (0_11919_01-122) is situated in a gene coding for a F-box protein, an enzyme link to the pathway of lignin precursor biosynthesis (Andersson Gunnerås 2005). This suggests that a putative F-box association to fiber width may be mediated through the control of lignin biosynthesis, an integral part of the secondary cell walls of plants. The SNP-QTL (Axs_47_502) significantly associated with GA is an UDP-D apiose/UDP-D-xylose synthase (AXS) that catalyzes the conversion of UDP-D-glucuronicacid to UDP-D-apiose and UDP-D-xylose. D-xylose is the second most common component of hemicellulose abundant in the cell wall, particularly in the S2 layer, which heavily influences the properties of the fiber cells (Rowell 2005). By theoretically investigating mechanical aspects of fiber cell division and maturation, Schulgasser and Witztum (2007) suggested the S2 microfibril angle (*i.e.*, MFA) to be involved in the development of grain angle. An association between GA and AXS observed in this study is thus conceivable.

Among the significant QTL for trait slopes, the SNP-QTL 2_10306_01-354 and 0_18350_01-393 were associated with LWD and FWr, respectively. The SNP associated with LWD is located in a gene coding the NINJA protein, which acts as a transcriptional repressor and whose activity is mediated by a functional TPL-binding EAR repression motif. Both NINJA and TPL proteins function as negative regulators of jasmonate responses and are therefore involved in the regulation of gene expression of proteins related to stress and growth (Wasternack and Hause 2013). The significant association of NINJA protein with WD in this study could be understood considering that wood density is a consequence of cell wall growth. The SNP associated with FWr is located in a gene coding a lipase class 3 family protein. Lipases are involved in the accumulation and storage of lipid triglycerides and steryl esters into an organelle in the cell called the lipid body (LB). Among other functions, LBs are involved in providing building blocks for the enlargement of cell membranes (van der Schoot *et al.* 2011), such as the plasma membrane of the cell walls that form the wood. These speculations on the nature of the significant SNPs control of certain traits will certainly require further research advances in the molecular biology of wood development to be confirmed. However, we do not doubt the value of our findings as candidate targets for such studies.

Previous studies of QTL and association analysis for wood properties resulted in the identification of multiple candidate genes, some of which have been confirmed across several studies in conifers (Brown *et al.* 2003; González-Martínez *et al.* 2007; Dillon *et al.* 2010; Beaulieu *et al.* 2011). The majority of these previously assayed candidate genes were included in our array, but we found no significant trait associations for them. The only exception is the finding made by Kumar *et al.* (2009) that a gene coding for a lipid transfer protein was differentially expressed between Scots pine juvenile and mature wood. This is consistent with the SNP located in the lipid transfer protein coding gene and associated with the slope of FWr in this study. The authors argued about a possible gene role on cell wall deposition but admitted that this interpretation was vague and required further research for its validation.

### Conclusions and future research

With respect to the genetic dissection of wood properties, the functional QTL mapping approach appeared promising and produced interesting results. Significant QTL were observed for the wood trait intercepts of EWD, FWr, and EFWr that appeared fairly stable within the studied cambial age range (approximately 5–13 yr). Two highly significant QTL were also detected for GA, which has never been included in wood property QTL studies. Furthermore, significant QTL were detected for the wood trait slopes of EP, EWD, LWD, and FWr, indicating marker associations with the rate of wood maturation. Four of the significant QTL were observed using SNP markers developed from candidate genes, thus making them interesting targets for further linkage or association mapping efforts or for molecular biology studies of the wood development in conifers.

In addition to the genetic implications of this study, two functional mapping methods and several methods for quantifying certainty or significance of QTL were compared. The one-step BLMM and two-step mLASSO methods detected similar sets of QTL given that a large number of individuals were studied and the adaptive shrinkage method of BLMM appeared to allow reasonably large effect estimates in case QTL were significant. When the number of individual studies was small, however, the BLMM method did not detect any QTL indicating greater requirements than mLASSO in terms of the number of assessed individuals. Future research in this regard could use simulated data to further elucidate the differences between the two functional mapping approaches.

## Supplementary Material

Supporting Information

## References

[bib1] Andersson Gunnerås, S., 2005 *Wood formation and transcript analysis with focus on tension wood and ethylene biology*. PhD dissertation, 2005:15, Swedish University of Agricultural Sciences, Umeå, Sweden.

[bib2] Beaulieu J. Doerksen T. Boyle B. Clément S. Deslauriers M., 2011 Association genetics of wood physical traits in the conifer white spruce and relationships with gene expression. Genetics 188: 197–214. 21385726 10.1534/genetics.110.125781 PMC3120141

[bib3] Beavis, W. D., 1994 The power and deceit of QTL experiments: lessons from comparative QTL-studies, pp 250–266 in *Proceedings of the 49^th^ Annual Corn and Sorghum Industry Research Conference*. American Seed Trade Association, Washington DC..

[bib4] Bouffier L. Raffin A. Rozenberg P. Meredieu C. Kremer A., 2009 What are the consequences of growth selection on wood density in the French maritime pine breeding programme? Tree Genet. Genomes 5: 11–25.

[bib5] Brendel O. Pot D. Plomion C. Rozenberg P. Guehl J. M., 2002 Genetic parameters and QTL analysis of δ^13^C and ring width in maritime pine. Plant Cell Environ. 25: 945–953.

[bib6] Brown G. R. Bassoni D. L. Gill G. P. Fontana J. R. Wheeler N. C., 2003 Identification of quantitative trait loci influencing wood property traits in loblolly pine (*Pinus taeda* L.). III. QTL verification and candidate gene mapping. Genetics 164: 1537–1546. 12930758 10.1093/genetics/164.4.1537 PMC1462646

[bib7] Burdon R. B. Kibblewhite R. P. Walker J. C. F. Megraw R. A. Evans R., 2004 Juvenile *vs.* mature wood: A new concept, orthogonal to corewood *vs.* outerwood, with special reference to *Pinus radiata* and *P. taeda*. For. Sci. 50: 399–415.

[bib8] Bühlmann P. Kalisch M. Meier L., 2014 High-dimensional statistics with a view towards applications in biology. Annu. Rev. Stat. Appl. 1: 255–278.

[bib9] Chatterjee A. Lahiri S. N., 2011 Bootstrapping LASSO estimators. J. Am. Stat. Assoc. 106: 608–625.

[bib10] Dillon S. K. Nolan M. Li W. Bell C. Wu H. X., 2010 Allelic variation in cell wall candidate genes affecting solid wood properties in natural populations and land races of *Pinus radiata*. Genetics 185: 1477–1487. 20498299 10.1534/genetics.110.116582 PMC2927771

[bib11] Dinwoodie J. M., 2000 Timber: Its Nature and Behaviour, Ed. 2 E & FN Spon, New York.

[bib12] Doyle J. Doyle J., 1990 Isolation of plant DNA from fresh tissue. Focus 12: 13–15.

[bib13] Efron B. Tibshirani R. Storey J. D. Tusher V., 2001 Empirical Bayes analysis of a microarray experiment. J. Am. Stat. Assoc. 96: 1151–1160.

[bib14] Emebiri L. C. Devey M. E. Matheson A. C. Slee M. U., 1997 Linkage of RAPD markers to NESTUR, a stem growth index in radiata pine seedings. Theor. Appl. Genet. 95: 119–124.

[bib15] Evans R., 1994 Rapid measurement of the transverse dimensions of tracheids in radial wood sections from *Pinus radiata*. Holzforschung 48: 168–172.

[bib16] Evans R., 2006 Wood stiffness by X-ray diffractometry, pp. 138–146 in Characterization of the Cellulosic Cell Wall, edited by Stokke D. D. Groom H. L. Wiley, Hoboken, NJ.

[bib17] Fahrmeir L. Kneib T., 2011 Bayesian Smoothing and Regression for Longitudinal, Spatial and Event History Data, Oxford University Press, New York.

[bib18] Feng C. Yuan Y. Wei W. Fan Z. Zhang T., 2012 Climatic response of ring width and maximum latewood density of *Larix sibirica* in the Altay Mountains, reveals recent warming trends. Ann. For. Sci. 69: 723–733.

[bib19] Friedman J. Hastie T. Tibshirani R., 2010 Regularization paths for generalized linear models via coordinate descent. J. Stat. Softw. 33: 1. 20808728 PMC2929880

[bib20] Fries A., 2012 Genetic parameters, genetic gain and correlated responses in growth, fibre dimensions and wood density in a Scots pine breeding population. Ann. For. Sci. 69: 783–794.

[bib21] Furlotte N. A. Eskin E. Eyheramendy S., 2012 Genome-wide association mapping with longitudinal data. Genet. Epidemiol. 36: 463–471. 22581622 10.1002/gepi.21640 PMC3625633

[bib22] Gapare W. J. Wu H. X. Abarquez A., 2006 Genetic control of the time of transition from juvenile to mature wood in *Pinus radiata* D. Don. Ann. For. Sci. 63: 871–878.

[bib23] Gapare W. J. Baltunis B. S. Ivkovíc M. Wu H. X., 2009 Genetic correlations among juvenile wood quality and growth traits and implications for selection strategy in *Pinus radiata* D. Don. Ann. For. Sci. 66: 606.

[bib24] Gaspar M. J. Louzada J. L. Aguiar A. Almeida A. M., 2008 Genetic correlations between wood quality traits of *Pinus pinaster* Ait. Ann. For. Sci. 65: 703.

[bib25] Gee C. Morrison J. L. Thomas D. C. Gauderman W. J., 2003 Segregation and linkage analysis for longitudinal measurements of a quantitative trait. BMC Genet. 4: S21. 14975089 10.1186/1471-2156-4-S1-S21 PMC1866456

[bib26] González-Martínez S. C. Wheeler N. C. Ersoz E. Nelson C. D. Neale D. B., 2007 Association genetics in *Pinus taeda* L. I. Wood property traits. Genetics 175: 399–409. 17110498 10.1534/genetics.106.061127 PMC1775017

[bib27] Grattapaglia D. Sederoff R., 1994 Genetic linkage maps of *Eucalyptus grandis* and *Eucalyptus urophylla* using a pseudo-testcross: mapping strategy and RAPD markers. Genetics 137: 1121–1137. 7982566 10.1093/genetics/137.4.1121 PMC1206059

[bib28] Grattapaglia D. Bertolucci F. L. Sederoff R. R., 1995 Genetic mapping of QTLs controlling vegetative propagation in *Eucalyptus grandis* and *E. urophylla* using a pseudo-testcross strategy and RAPD markers. Theor. Appl. Genet. 90: 933–947. 24173047 10.1007/BF00222906

[bib29] Green H. V. Worral J., 1964 Wood quality studies I. A scanning microphometer for automatically measuring and recording certain wood characteristics. Tappi. 47: 419–427.

[bib30] Guan Y. Stephens M., 2011 Bayesian variable selection regression for genome-wide association studies, and other large-scale problems. Ann. Appl. Stat. 5: 1780–1815.

[bib31] Habier D. Fernando R. L. Kizilkaya K. Garrick D. J., 2011 Extension of the Bayesian alphabet for genomic selection. BMC Bioinformatics 12: 186. 21605355 10.1186/1471-2105-12-186 PMC3144464

[bib32] Hallingbäck H. R. Jansson G. Hannrup B. Fries A., 2010 Which annual rings to assess grain angles in breeding of Scots pine for improved shape stability of sawn timber? Silva Fenn. 44: 275–288.

[bib33] Hannrup B. Ekberg I. Persson A., 2000 Genetic correlations among wood, growth capacity and stem traits in *Pinus sylvestris*. Scand. J. For. Res. 15: 161–170.

[bib34] Hannrup B. Säll H. Jansson G., 2003 Genetic parameters for spiral grain in Scots pine and Norway spruce. Silvae Genet. 52: 215–220.

[bib35] Hastie, T., R. Tibshirani, and J. Friedman, 2009 *Elements of Statistical Learning*, Ed. 2. Springer: New York..

[bib36] Heuven, H. C. M., and L. L. G. Janss, 2010 Bayesian multi-QTL mapping for growth curve parameters. BMC Proceedings 4: S12. 10.1186/1753-6561-4-s1-s12 PMC2857843 20380755

[bib37] Hong Z. Fries A. Wu H. X., 2014 High negative genetic correlations between growth traits and wood properties suggest incorporating multiple traits selection including economic weights for the future Scots pine breeding programs. Ann. For. Sci. 71: 463–472.

[bib38] Hurtado P. X. Schnabel S. K. Zaban A. Veteläinen M. Virtanen E., 2011 Dynamics of senescence-related QTLs in potato. Euphytica 183: 289–302.

[bib39] Hägglund, B., and J.-E. Lundmark, 1982 *Handledning i bonitering med skogshögskolans boniteringssystem*. National board of forestry, Jönköping, Sweden. ISBN 9185748641.

[bib40] Kao C.-H. Zeng Z.-B. Teasdale D., 1999 Multiple interval mapping for quantitative trait loci. Genetics 152: 1203–1216. 10388834 10.1093/genetics/152.3.1203 PMC1460657

[bib41] Kaya Z. Sewell M. M. Neale D. B., 1999 Identification of quantitative trait loci influencing annual height- and diameter-increment growth in loblolly pine (*Pinus taeda L*.). Theor. Appl. Genet. 98: 586–592.

[bib42] Knott S. A. Neale D. B. Sewell M. M. Haley C. S., 1997 Multiple marker mapping of quantitative trait loci in an outbred pedigree of loblolly pine. Theor. Appl. Genet. 94: 810–820.

[bib43] Kujala S. T. Savolainen O., 2012 Sequence variation patterns along a latitudinal cline in Scots pine (*Pinus sylvestris*): signs of clinical adaptation? Tree Genet. Genomes 8: 1455–1467.

[bib44] Kumar S. Spelman R. J. Garrick D. J. Richardson T. E. Lausberg M., 2000 Multiple-marker mapping of wood density loci in an outbred pedigree of radiata pine. Theor. Appl. Genet. 100: 926–933.

[bib45] Kumar M. Saranpää P. Barnett J. R. Wilkinson M. J., 2009 Juvenile-mature wood transition in pine: correlation between wood properties and candidate gene expression profiles. Euphytica 166: 341–355.

[bib46] Lander E. S. Botstein D., 1989 Mapping Mendelian factors underlying quantitative traits using RFLP linkage maps. Genetics 121: 185–199. 2563713 10.1093/genetics/121.1.185 PMC1203601

[bib47] Lerceteau E. Szmidt A. E., 2000 AFLP mapping and detection of quantitative trait loci (QTLs) for economically important traits in *Pinus sylvestris*: a preliminary study. Mol. Breed. 6: 451–458.

[bib48] Lerceteau E. Szmidt A. E. Andersson B., 2001 Detection of quantitative trait loci in *Pinus sylvestris* L. across years. Euphytica 121: 117–122.

[bib49] Li Z. Sillanpää M. J., 2012 Overview of LASSO-related penalized regression methods for quantitative trait mapping and genomic selection. Theor. Appl. Genet. 125: 419–435. 22622521 10.1007/s00122-012-1892-9

[bib50] Li Z. Sillanpää M. J., 2013 A Bayesian nonparametric approach for mapping dynamic quantitative traits. Genetics 194: 997–1016. 23770698 10.1534/genetics.113.152736 PMC3730925

[bib51] Lin M. Wu R., 2006 A joint model for nonparametric functional mapping of longitudinal trajectory and time-to-event. BMC Bioinformatics 7: 138. 16539724 10.1186/1471-2105-7-138 PMC1479376

[bib52] Lockhart R. Taylor J. Tibshirani R. J. Tibshirani R., 2014 A significance test for the LASSO. Ann. Stat. 42: 413–468. 10.1214/13-AOS1175 PMC4285373 25574062

[bib53] Ma C. Casella G. Wu R., 2002 Functional mapping of quantitative trait loci underlying the character process: a theoretical framework. Genetics 161: 1751–1762. 12196415 10.1093/genetics/161.4.1751 PMC1462199

[bib54] Ma C. Lin M. Littell R. C. Yin T. Wu R., 2004 A likelihood approach for mapping growth trajectories using dominant markers in a phase-unknown full-sib family. Theor. Appl. Genet. 108: 699–705. 14586505 10.1007/s00122-003-1484-9

[bib55] Markussen T. Fladung M. Achere V. Favre J. M. Faivre-Rampant P., 2003 Identification of QTLs controlling growth, chemical and physical wood property traits in *Pinus pinaster* (Ait.). Silvae Genet. 52: 8–15.

[bib56] Meinshausen N. Meier L. Bühlmann P., 2009 P-Values for high-dimensional regression. J. Am. Stat. Assoc. 104: 1671–1681.

[bib57] Meinshausen N. Bühlmann P., 2010 Stability Selection. J. R. Stat. Soc. Series B. 72: 417–473.

[bib58] Mork E., 1928 Die qualität des Fichtenholzes unter befonderer Rückfichtnahme auf Schleif—und Papierholz. Der Papier-Fabrikant. 26: 741–747.

[bib59] Mullin, T., B. Andersson, J.-C. Bastien, J. Beaulieu, R. D. Burdon *et al.*, 2011 Chapter 2: Economic Importance, Breeding Objectives and Achievements, pp. 40–127 in *Genetics*, *Genomics and Breeding of Conifers*, edited by C. Plomion, J. Bousquet, and C. Kole. Science Puslishers, Inc., Enfield, NH, Edenbridge Ltd, UK.

[bib60] Olsson, L., 2000 Measurement of Latewood—Comparison of different methods and definitions, *Third Workshop of COST Action E10 Wood Properties for Industrial Use*. Helsinki, Finland, June 19–21,2000.

[bib61] Palmé A. E. Wright M. Savolainen O., 2008 Patterns of divergence among Conifer ESTs and polymorphism in *Pinus sylvestris* identify putative selective sweeps. Mol. Biol. Evol. 25: 2567–2577. 18775901 10.1093/molbev/msn194

[bib62] Piepho H. P. Moehring J. Schulz-Streeck T. Ogutu J. O., 2012 A stage-wise approach for analysis of multi-environment trials. Biom. J. 54: 844–860. 23007738 10.1002/bimj.201100219

[bib63] Plomion C. Durel C.-E. O’Malley D., 1996 Genetic dissection of height in maritime pine seedlings raised under accelerated growth condition. Theor. Appl. Genet. 93: 849–858. 24162417 10.1007/BF00224085

[bib64] Pot D. Rodrigues J. C. Rozenberg P. Chantre G. Tibbits J., 2006 QTLs and candidate genes for wood properties in maritime pine (*Pinus pinaster* Ait.). Tree Genet. Genomes 2: 10–24.

[bib65] Pyhäjärvi T. García-Gil M. R. Knürr T. Mikkonen M. Wachowiak W., 2007 Demographic history has influenced nucleotide diversity in European *Pinus sylvestris* populations. Genetics 177: 1713–1724. 18039881 10.1534/genetics.107.077099 PMC2147978

[bib66] Pyhäjärvi T. Kujala S. T. Savolainen O., 2011 Revisiting protein heterozygosity in plants-nucleotide diversity in allozyme coding genes of conifer *Pinus sylvestris*. Tree Genet. Genomes 7: 385–397.

[bib67] Rosvall O, 2011 Review of the Swedish Tree Breeding Programme, Stiftelsen Skogsbrukets Forskninsinstitut, Uppsala, Sweden.

[bib68] Rowell R. M., 2005 Handbook of Wood Chemistry and Wood Composites, CRC Press, Boca Raton, Florida.

[bib69] Schulgasser K. Witztum A., 2007 The mechanism of spiral grain formation in trees. Wood Sci. Technol. 41: 133–156.

[bib70] Scott J. G. Berger J. O., 2010 Bayes and empirical-Bayes multiplicity adjustment in the variable-selection problem. Ann. Stat. 38: 2587–2619.

[bib71] Segura V. Vilhjálmsson B. J. Platt A. Korte A. Seren Ü., 2012 An efficient multi-locus mixed-model approach for genome-wide association studies in structured populations. Nat. Genet. 44: 825–830. 22706313 10.1038/ng.2314 PMC3386481

[bib72] Sewell M. M. Bassoni D. L. Megraw R. A. Wheeler N. C. Neale D. B., 2000 Identification of QTLs influencing wood property traits in loblolly pine (*Pinus taeda* L.). I. Physical wood properties. Theor. Appl. Genet. 101: 1273–1281. 10.1007/s001220100697 12582689

[bib73] Sewell M. M. Davis M. F. Tuskan G. A. Wheeler N. C. Elam C. C., 2002 Identification of QTLs influencing wood property traits in loblolly pine (*Pinus taeda* L.). II. Chemical wood properties. Theor. Appl. Genet. 104: 214–222. 12582689 10.1007/s001220100697

[bib74] Sillanpää M. J. Pikkuhookana P. Abrahamsson S. Knürr T. Fries A., 2012 Simultaneous estimation of multiple quantitative trait loci and growth curve parameters through hierarchical Bayesian modeling. Heredity 108: 134–146. 21792229 10.1038/hdy.2011.56 PMC3262873

[bib75] Sikorska K. Rivadeneira F. Groenen P. J. F. Hofman A. Uitterlinden A. G., 2012 Fast linear mixed model computations for genome-wide association studies with longitudinal data. Stat. Med. 32: 165–180. 22911890 10.1002/sim.5517

[bib76] Slate J., 2013 From Beavis to beak color: a simulated study to examine how much QTL mapping can reveal about the genetic architecture of quantitative traits. Evolution 67: 1251–1262. 23617906 10.1111/evo.12060

[bib77] Snedecor G. W. Cochran W. G., 1980 Statistical Methods, Ed. 7 The Iowa State University Press, Ames, Iowa.

[bib78] Tibshirani R., 1996 Regression shrinkage and selection via the lasso. J. R. Stat. Soc., B 58: 267–288.

[bib79] Ukrainetz N. K. Ritland K. Mansfield S. D., 2008 Identification of quantitative trait loci for wood quality and growth across eight full-sib coastal Douglas-fir families. Tree Genet. Genomes 4: 159–170.

[bib80] van der Schoot C. Paul L. K. Paul S. B. Rinne P. L. H., 2011 Plant lipid bodies and cell-cell signaling: A new role for an old organelle? Plant Signal. Behav. 6: 1732–1738. 22057325 10.4161/psb.6.11.17639 PMC3329345

[bib81] Ventrucci M. Scott E. M. Cocchi D., 2011 Multiple testing on standardized mortality ratios: a Bayesian hierarchical model for FDR estimation. Biostatistics 12: 51–67. 20577014 10.1093/biostatistics/kxq040

[bib82] Vos P. Hogers R. Bleeker M. Reijans M. van de Lee T., 1995 AFLP - a new technique for DNA-fingerprinting. Nucleic Acids Res. 23: 4407–4414. 7501463 10.1093/nar/23.21.4407 PMC307397

[bib83] Wang, T., 2012 Linear mixed effects model for a longitudinal genome wide association study of lipid measures in type 1 diabetes. PhD thesis, McMaster University, Hamilton, Canada.

[bib84] Warensjö M. Rune G., 2004 Effects of compression wood and grain angle on deformations of studs from 22-year old Scots pine trees. Scand. J. For. Res. 19(Suppl 5): 48–54.

[bib85] Wasternack C. Hause B., 2013 Jasmonates: biosynthesis, perception, signal transduction and action in plant stress response, growth and development. An update to the 2007 review. Ann. Bot. (Lond.) 111: 1021–1058. 10.1093/aob/mct067 PMC3662512 23558912

[bib86] Weiss R. E., 2005 Modeling Longitudinal Data, Springer, New York.

[bib87] Wu R. L. Ma C. X. Yang M. C. K. Chang M. Santra U., 2003 Quantitative trait loci for growth in *Populus*. Genet. Res. 81: 51–64. 12693683 10.1017/s0016672302005980

[bib88] Wu, R. L., and M. Lin, 2006 Functional mapping—A new tool to study the genetic architecture of dynamic complex traits. Nat. Rev. Genet. **7:** 229–237. 10.1038/nrg1804 16485021

[bib89] Xu S., 2003 Estimating polygenic effects using markers of the entire genome. Genetics 163: 789–801. 12618414 10.1093/genetics/163.2.789 PMC1462468

[bib90] Xu S., 2007 An empirical Bayes method for estimating epistatic effects of quantitative trait loci. Biometrics 63: 513–521. 17688503 10.1111/j.1541-0420.2006.00711.x

[bib91] Yang R. Xu S., 2007 Bayesian shrinkage analysis of quantitative trait loci for dynamic traits. Genetics 176: 1169–1185. 17435239 10.1534/genetics.106.064279 PMC1894582

[bib92] Zeng Z.-B., 1994 Precision mapping of quantitative trait loci. Genetics 136: 1457–1468. 8013918 10.1093/genetics/136.4.1457 PMC1205924

[bib93] Zobel B. J. Sprague J. R., 1998 Juvenile Wood in Forest Trees, Springer, Berlin.

