# Supplementary material for: Functional Multi-Locus QTL Mapping of Temporal Trends in Scots Pine Wood Traits
Source: G3 (Bethesda). 2014 Oct 9;4(12):2365–79. doi: 10.1534/g3.114.014068 (PMC4267932; doi:10.1534/g3.114.014068)
Supplement: Supporting Information [file supp_4_12_2365__index.html]

Functional Multi-Locus QTL Mapping of Temporal Trends in Scots Pine Wood Traits — Supporting Information 

# Functional Multi-Locus QTL Mapping of Temporal Trends in Scots Pine Wood Traits

## Supporting Information for Li *et al.*, 2014

**Files in this Data Supplement:**

- File S5 - Supporting Information (PDF, 294 KB)
- File S1 - Silviscan wood property measurements for 286 individuals. (.xlsx, 384 KB)
- File S2 - Grain angle (GA) measurements for 492 individuals. (.xlsx, 19 KB)
- File S3 - Genotype dataset for the 637 SNPs (out of 768) that were successfully amplified and genotyped for 91 offspring plus 2 parents. (.xlsx, 258 KB)
- File S4 - Genotype dataset for the 508 AFLPs that were genotyped for 497 offspring individuals plus 2 parents. (.xlsx, 872 KB)
