# Supplementary material for: Functional Multi-Locus QTL Mapping of Temporal Trends in Scots Pine Wood Traits
Source: G3 (Bethesda). 2014 Oct 9;4(12):2365–79. doi: 10.1534/g3.114.014068 (PMC4267932; doi:10.1534/g3.114.014068)
Supplement: Supporting Information [file supp_g3.114.014068_FileS5.pdf]

Supporting Information

Functional multi-locus QTL mapping of temporal trends in Scots pine wood traits

Zitong Li, Henrik R. Hallingbäck, Sara Abrahamsson, Anders Fries, Bengt Andersson Gull, Mikko J. Sillanpää, M Rosario García-Gil

1 Marker mapping supplementary

Marker sorting and mapping was performed with all the available genotype data simultaneously with the aim of constructing one pure AFLP dataset (A-set) for the larger subset of individuals and one mixed SNP-AFLP dataset (S+A-set) intended for the smaller subset. Because the studied full-sib family was generated by two non-inbred and highly heterozygotic parents, a two-way pseudo-testcross mapping strategy was employed (e.g. Grattapaglia and Sederoff, 1994; Grattapaglia et al., 1995). Markers for which genotyping scoring success was inadequate ($< 80\%$) and poorly genotyped individuals ($< 70\%$) were excluded from further study. By checking parental heterozygosity and genotypic frequencies of the offspring, an additional selection was performed where codominant SNP markers were accepted given that genotypes segregated according to 1:1 proportions (one of the parents being heterozygote) or to 1:2:1 proportions (both parents heterozygotic). For dominant AFLP however, only a 1:1 band presence/absence segregation proportion was accepted (only one parent heterozygote). Selection was performed based on submitting genotype proportions to χ^2-tests using 0.01 as a threshold for the p-value.

Among the selected markers, recombination fractions were calculated for all pairs of markers in order to estimate marker linkage and phase. For most marker pairs this could be done by division, but for pairs of 1:2:1-segregating SNPs, recombination frequencies were estimated via the maximum likelihood EM-algorithm (Dempster et al., 1977). Linkage between markers was declared based on a log-likelihood ratio of 23.03 or higher (equivalent to LOD>5). Even though the inclusion of 1:2:1-segregating markers made the construction of a consensus map possible, it was nonetheless reasonable to assume that different sets of QTL segregated within each of the unrelated and heterozygotic parents and it was thus more meaningful to separate the marker linkage mapping into maternal and paternal sections. The 1:1-segregating markers were assigned to linkage groups based on the parent for which they exhibited heterozygosity as well as on linkage. The 1:2:1-segregating markers were instead duplicated into two identical 1:1-segregating markers each assigned to male and female linkage groups respectively. Heterozygote genotypes of 1:2:1-segregating markers were henceforth treated as unknown (replaced by missing

values). Consequently all marker genotypes were recoded as numeric values of 0, 1 or missing value.

Genetic distances between markers within each linkage group were estimated using the Haldane mapping function and markers were then ordered by minimizing the sum of adjacent genetic distances using the branch-and-bound searching algorithm. Preliminary analyses showed that genetic distances estimated using the more common Kosambi mapping function behaved in a less additive manner than the Haldane function and therefore the latter was used. Markers that were strongly correlated ($|r| > 0.95$ approximately equivalent to a 2.5 cM genetic distance) to another marker with a better genotype coverage were excluded from further analysis due to redundancy.

In summary 153 AFLP markers genotyped on 455 individuals (the A-dataset) and 153 AFLP and 166 SNP markers genotyped on 91 individuals (the S+A-dataset) were retained in the analysis after filtering and sorting. 251 markers were distributed on 26 maternal and 24 paternal linkage groups while 68 markers could not be assigned to any linkage group (unclustered).

By cross-examining the linkages of the duplicated 1:2:1-segregating SNPs, 15 maternal linkage groups were successfully associated with the corresponding paternal linkage groups. Missing genotype values were imputed by their conditional expectation estimated from flanking markers with known genotypes in accordance with Haley and Knott (1992), but taking marker phase (coupling/repulsion) into account. The imputation of *missing heterozygote* values for the duplicated 1:2:1-segregating markers was conducted by selecting the marker duplicate that exhibited the closest linkage with flanking markers and imputing its missing values conventionally (x_1). Subsequently, the missing values of the second marker duplicate located in the linkage group of the opposite sex, were imputed using the opposite values of the first duplicate imputation ($x_2 = 1 - x_1$). Using this mirror imputation technique it was ensured that the separation of 1:2:1-segregating markers into pairs of 1:1-segregating markers was made as complete as possible and to break down the artefactual correlation between duplicated markers.

2 The Bayesian liner mixed effects model

2.1 Gibbs sampling algorithm

The longitudinal linear mixed effect model (LMM) is

$$y_{ik} = \alpha_0 + \alpha_1 t_{ik} + \alpha_{i0} + \alpha_{i1} t_{ik} + \sum_{j=1}^{p} x_{ij}\beta_j + \sum_{j=1}^{p} x_{ij} t_{ik}\gamma_j + \varepsilon_{ik}, \qquad \varepsilon_{ik} \overset{\text{i.i.d.}}{\sim} N(0, \sigma_0^2), \tag{2.1}$$

for $i = 1, ..., n$, $k = 1, ..., m_i$, and $j = 1, ..., p$. Equation (2.1) can be written in a vector/matrix form:

$$\boldsymbol{y}_i = \boldsymbol{W}_i\boldsymbol{\alpha} + \boldsymbol{W}_i\boldsymbol{\alpha}_i + \sum_{j=1}^{2p} \boldsymbol{z}_{ij} B_j + \boldsymbol{\varepsilon}_i, \qquad \boldsymbol{\varepsilon}_i \overset{\text{i.i.d.}}{\sim} \text{MVN}(\boldsymbol{0}, \sigma_0^2 \boldsymbol{I}_{m_i \times m_i}), \tag{2.2}$$

where $\boldsymbol{y}_i = [y_{i1}, ..., y_{im_i}]'$, $\boldsymbol{W}_i = \begin{pmatrix} 1 & t_{i1} \\ \vdots & \vdots \\ 1 & t_{im_i} \end{pmatrix}$, $\boldsymbol{\alpha} = [\alpha_0, \alpha_1]'$, $\boldsymbol{\alpha}_i = [\alpha_{i0}, \alpha_{i1}]'$,

$$\boldsymbol{z}_{ij} = \begin{cases} \overbrace{[x_{ij}, ..., x_{ij}]'}^{m_i \text{ times}}, & \text{for } j = 1, ..., p \\ [x_{ij-p}t_{i1}, ..., x_{ij-p}t_{im_i}]', & \text{for } j = p+1, ..., 2p \end{cases}, \text{ and } B_j = \begin{cases} \beta_j, & \text{for } j = 1, ..., p \\ \gamma_{j-p}, & \text{for } j = p+1, ..., 2p \end{cases}.$$

Equation (2.2) can be written as the following likelihood function

$$p(\boldsymbol{Y}|\boldsymbol{\theta}) = (2\pi\sigma^2)^{-\frac{N}{2}} \exp[-\frac{\sum_{i=1}^{n}(\boldsymbol{y}_i - \boldsymbol{W}_i\boldsymbol{\alpha} - \boldsymbol{W}_i\boldsymbol{\alpha}_i - \sum_{j=1}^{2p}\boldsymbol{z}_{ij}B_j)'(\boldsymbol{y}_i - \boldsymbol{W}_i\boldsymbol{\alpha} - \boldsymbol{W}_i\boldsymbol{\alpha}_i - \sum_{j=1}^{2p}\boldsymbol{z}_{ij}B_j)}{2\sigma^2}], \tag{2.3}$$

where $N = \sum_{i=1}^{n} m_i$, $\boldsymbol{\theta}$ represents all the parameters in the model. Furthermore, the priors are

- $p(\alpha_0) \propto 1_{(-\infty,\infty)}$,
- $p(\alpha_1) \propto 1_{(-\infty,\infty)}$,
- $p(\sigma^2) \propto \frac{1}{\sigma^2}$,
- $p(\boldsymbol{\alpha}_i|\boldsymbol{\Sigma}_{2\times2}) = \text{MVN}(\boldsymbol{\alpha}_i|\boldsymbol{0}, \boldsymbol{\Sigma}_{2\times2})$, for $i = 1, ..., n$,
- $p(\boldsymbol{\Sigma}_{2\times2}) = \text{Inv-Wishart}(\boldsymbol{\Sigma}_{2\times2}|\boldsymbol{\Psi}_{2\times2}, v)$,
- $p(B_j) = (1 - r_j)1_{\{B_j=0\}} + r_j N(B_j|0, \sigma_j^2)$, for $j = 1, ..., 2p$,
- $p(r_j) = w^{r_j}(1 - w)^{1-r_j}$
- $p(\sigma_j^2) = \text{Inv-Gamma}(\sigma_j^2|a, b)$,

where $\boldsymbol{\Psi}_{2\times2} = \boldsymbol{I}_{2\times2}$, $v = 1$, $a = b = 0.1$, and $w = 0.5$.

In Bayesian statistics, our target is the posterior distribution $p(\boldsymbol{\theta}|\boldsymbol{Y}) \propto p(\boldsymbol{Y}|\boldsymbol{\theta})p(\boldsymbol{\theta})$, proportional to the product of the likelihood function and priors. We use the Gibbs sampling method (Geman and Geman, 1984), a Markov Chain Monte Carlo (MCMC) algorithm, to evaluate the full posterior distribution. The following is a skeleton of the Gibbs sampler:

i) assign an initial state of the unknown parameters $\boldsymbol{\theta}^0 = \theta_1^0, ..., \theta_M^0$ (M represents the total number of all the single parameters).

ii) For $j = 1, ..., M$: sample each parameter θ_j^1 from its full conditional posterior distribution $p(\theta_j|\theta_1^1, ..., \theta_{j-1}^1, \theta_{j+1}^0, ..., \theta_M^0, \boldsymbol{Y})$.

iii) Repeat step (2) by simulating $\boldsymbol{\theta}^2$, $\boldsymbol{\theta}^3$,... for many times.

Note that during step (2), it is also possible to update a group of parameters as a block, if it is possible to sample from their joint full conditional posterior distribution.

Next, all the required full conditional posterior distributions for the LMM model are provided.

(I) The full conditional posterior of $\boldsymbol{\alpha}$: $p(\boldsymbol{\alpha}|\boldsymbol{\theta}_{-\boldsymbol{\alpha}}, \boldsymbol{Y}) = \text{MVN}(\boldsymbol{\alpha}|\boldsymbol{\mu}_{\boldsymbol{\alpha}}, \boldsymbol{\Sigma}_{\boldsymbol{\alpha}})$ with

$$\boldsymbol{\mu}_{\boldsymbol{\alpha}} = \frac{1}{n}\sum_{i=1}^{n}(\boldsymbol{W}_i'\boldsymbol{W}_i)^{-1}\boldsymbol{W}_i'(\boldsymbol{y}_i - \boldsymbol{W}_i\boldsymbol{\alpha}_i - \sum_{j=1}^{2p}\boldsymbol{z}_{ij}B_j)'(\boldsymbol{y}_i - \boldsymbol{W}_i\boldsymbol{\alpha}_i - \sum_{j=1}^{2p}\boldsymbol{z}_{ij}B_j), \quad (2.4)$$

and

$$\boldsymbol{\Sigma}_{\boldsymbol{\alpha}} = \sigma^2\sum_{i=1}^{n}(\boldsymbol{W}_i'\boldsymbol{W}_i)^{-1}. \quad (2.5)$$

(II) The full conditional posterior of $\boldsymbol{\alpha}_i$ (for $i = 1, ..., n$): $p(\boldsymbol{\alpha}_i|\boldsymbol{\theta}_{-\boldsymbol{\alpha}_i}, \boldsymbol{Y}) = \text{MVN}(\boldsymbol{\alpha}_i|\boldsymbol{\mu}_{\boldsymbol{\alpha}_i}, \boldsymbol{\Sigma}_{\boldsymbol{\alpha}_i})$ with

$$\boldsymbol{\mu}_{\boldsymbol{\alpha}} = (\boldsymbol{W}_i'\boldsymbol{W}_i)^{-1}\boldsymbol{W}_i'(\boldsymbol{y}_i - \boldsymbol{W}_i\boldsymbol{\alpha} - \sum_{j=1}^{2p}\boldsymbol{z}_{ij}B_j)'(\boldsymbol{y}_i - \boldsymbol{W}_i\boldsymbol{\alpha} - \sum_{j=1}^{2p}\boldsymbol{z}_{ij}B_j), \quad (2.6)$$

and

$$\boldsymbol{\Sigma}_{\boldsymbol{\alpha}_i} = \sigma^2(\boldsymbol{W}_i'\boldsymbol{W}_i)^{-1}. \quad (2.7)$$

(III) The full conditional posterior of B_j and r_j (for $j = 1, ..., 2p$): Parameters B_j and r_j are sampled as a pair in order to guarantee the sampler is mixing well (Geman and Geman, 1984; Titsias and Läzaro-Gredilla, 2011). Note that $p(B_j, r_j|\boldsymbol{\theta}_{-[B_j,r_j]}, \boldsymbol{Y}) = p(r_j|\boldsymbol{\theta}_{-[B_j,r_j]}, \boldsymbol{Y})$
$p(B_j|r_j, \boldsymbol{\theta}_{-[B_j,r_j]}, \boldsymbol{Y})$. Thus, we can first sample r_j from $p(r_j|\boldsymbol{\theta}_{-[B_j,r_j]}, \boldsymbol{Y})$, and then sample B_j from $p(B_j|r_j, \boldsymbol{\theta}_{-[B_j,r_j]}, \boldsymbol{Y})$. The required distributions are derived as follows:

- $p(r_j = 1|\boldsymbol{\theta}_{-[B_j,r_j]}, \boldsymbol{Y}) = \int p(r_j = 1, B_j|\boldsymbol{\theta}_{-[B_j,r_j]}, \boldsymbol{Y})dB_j = \frac{wE}{wE+1-w}$, with

$$E = \frac{\sigma_{B_j}}{\sigma_j}\exp(\frac{\mu_{B_j}}{2\sigma^2_{B_j}}), \quad (2.8)$$

$$\mu_{B_j} = \frac{\sigma^2_{B_j}}{\sigma^2}\sum_{i=1}^{n}\boldsymbol{z}_{ij}'(\boldsymbol{y}_i - \boldsymbol{W}_i\boldsymbol{\alpha} - \boldsymbol{W}_i\boldsymbol{\alpha}_i - \sum_{k\neq j}\boldsymbol{z}_{ik}B_k), \quad (2.9)$$

and

$$\sigma^2_{B_j} = \frac{\sigma^2}{\sum_{i=1}^{n}\boldsymbol{z}_i'\boldsymbol{z}_i + \frac{\sigma^2}{\sigma_j^2}}, \quad (2.10)$$

- $p(r_j = 0|\boldsymbol{\theta}_{-[B_j,r_j]}, \boldsymbol{Y}) = 1 - p(r_j = 1|\boldsymbol{\theta}_{-[B_j,r_j]}, \boldsymbol{Y})$,
- $p(B_j|r_j = 1, \boldsymbol{\theta}_{-[B_j,r_j]}, \boldsymbol{Y}) = N(B_j|\mu_{B_j}, \sigma^2_{B_j})$, and

- $p(B_j|r_j = 0, \boldsymbol{\theta}_{-[B_j,r_j]}, \boldsymbol{Y}) = 1_{\{B_j=0\}}$. By other mean, when $r_j = 0$, we have $B_j = 0$.

(IV) The full conditional posterior of σ^2: $p(\sigma^2|\boldsymbol{\theta}_{-\sigma^2}, \boldsymbol{Y}) = \text{Inv-Gamma}(\sigma^2|a_1, b_1)$ with $a_1 = \frac{N}{2}$, and $b_1 = \frac{\sum_{i=1}^n(\boldsymbol{y}_i - \boldsymbol{W}_i\boldsymbol{\alpha} - \boldsymbol{W}_i\boldsymbol{\alpha}_i - \sum_{j=1}^{2p}\boldsymbol{z}_{ij}B_j)'(\boldsymbol{y}_i - \boldsymbol{W}_i\boldsymbol{\alpha} - \boldsymbol{W}_i\boldsymbol{\alpha}_i - \sum_{j=1}^{2p}\boldsymbol{z}_{ij}B_j)}{2}$.

(V) The full conditional posterior of σ_j^2 (for $j = 1, ..., 2p$): $p(\sigma_j^2|\boldsymbol{\theta}_{-\sigma_j^2}, \boldsymbol{Y}) = \text{Inv-Gamma}(\sigma_j^2|a_2, b_2)$ with $a_2 = 0.5r_j + a$ and $b_2 = 0.5B_j^2 + b$.

(VI) The full conditional posterior of $\boldsymbol{\Sigma}_{2\times2}$: $p(\boldsymbol{\Sigma}_{2\times2}|\boldsymbol{\theta}_{-\boldsymbol{\Sigma}_{2\times2}},, \boldsymbol{Y}) = \text{Inv-Wishart}(\boldsymbol{\Sigma}_{2\times2}|\boldsymbol{\Phi}, \tau)$ with $\boldsymbol{\Phi} = \sum_{i=1}^n \boldsymbol{W}_i'\boldsymbol{W}_i + \boldsymbol{\Psi}_{2\times2}$ and $\tau = n + v$.

2.2 Posterior summarization

For each data set, we all-together simulated 50000 MCMC samples from the Gibbs sampler, by using the first 10000 were considered as burn-in, the remaining were stored in every 20th, so that eventually we obtained 2000 MCMC samples. Those samples provide an approximation to the full posterior distribution. The samples of a single variable is an approximation to its marginal posterior distribution. The posterior mean (the arithmetic mean of 2000 MCMC samples) $\hat{\theta}$ can be considered as the point estimate of each single parameter θ. Specially, we calculate the posterior mean of $p(r_j = 0|\boldsymbol{\theta}_{-[B_j,r_j]}, \boldsymbol{Y})$ instead of posterior mean of r_j, and consider $\hat{p}(r_j = 0|\boldsymbol{\theta}_{-[B_j,r_j]}, \boldsymbol{Y})$ as an approximation to the posterior inclusion probability of marker j ($j = 1, ..., p$). Based on the Rao-Blackwell theorem, this treatment helps to reduce the sample variance (Guan and Stephens, 2011).

References

Dempster, A. P., N. M. Laird and D. B. Rubin, 1977. Likelihood from incomplete data via the EM algorithm. Journal of the Royal Statistical Society: Series B. 39: 1–38.

Geman, S., and D. Geman, 1984. Stochastic relaxation, gibbs distributions, and the bayesian restoration of images. IEEE Transactions on Pattern Analysis and Machine Intelligence. 6: 721–741.

Grattapaglia, D. and R. Sederoff, 1994. Genetic linkage maps of Eucalyptus grandis and Eucalyptus urophylla using a pseudo-testcross: mapping strategy and RAPD markers. Genetics. 137: 1121–1137.

Grattapaglia, D., F. L. Bertolucci and R. R. Sederoff 1995. Genetic mapping of QTLs controlling vegetative propagation in Eucalyptus grandis and E. urophylla using a pseudo-testcross strategy and RAPD markers. Theoretical and Applied Genetics. 90: 933–947.

Guan, Y., and M. Stephens, 2011. Bayesian variable selection regression for genome-wide association studies, and other large-scale problems. Annals of Applied Statistics. 5: 1780–1815.

Haley, C. S. and S. A. Knott 1992. A simple regression method for mapping quantitative loci in line crosses using flanking markers. Heredity. 69: 315–324.

Kuo, L., and B. Mallick, 1998. Variable selection for regression models. Sankhya. B 60: 65–81.

Titsias, M., and M. Läzaro-Gredilla, 2011. Spike and slab variational inference for multi-task and multiple kernel learning. In Advances in neural information processing systems. 24: 2339–2347.
